# Supplementary material for: Multiplex Detection of 24 Staphylococcal Enterotoxins in Culture Supernatant Using Liquid Chromatography Coupled to High-Resolution Mass Spectrometry
Source: Toxins (Basel). 2022 Mar 31;14(4):249. doi: 10.3390/toxins14040249 (PMC9031063; doi:10.3390/toxins14040249)
Supplement: Supplementary file 1 [file toxins-14-00249-s001.zip › toxins-1652496-supplementary.pdf]

Supplementary Materials: Multiplex detection of 24 staphylococcal enterotoxins in culture supernatant using Liquid Chromatography coupled to High Resolution Mass Spectrometry.

Donatien Lefebvre, Kevin Blanco-Valle, Jacques-Antoine Hennekinne, Stéphanie Simon, François Fenaille, François Becher and Yacine Nia

Table S1. Origin of *Staphylococcus aureus* strains.

Strain	Accession no.	sample origin	Bacteria count (cfu/g)	Related publication	SE detected in food (official method)	Date of isolation	Geographical location	Strain origin
05CEB51		Rice	NA(1)	/	NA	2005	France	SFPO(2)
05CEB52	ERS4338875	Human	NA	Merda et al, 2020	NA	2005	France	SFPO
05CEB53		Human	NA	/	NA	2005	France	SFPO
06CEB196		Coconut beads	NA	/	Vidas: positive	2006	France	SFPO
06CEB83		Cheese	NA	/	Vidas: positive	2006	France	Own Check
08CEB393		Wrap salmon	NA	/	Vidas: positive	2008	France	SFPO
08CEB402		Blood sausage	NA	/	Vidas: positive	2008	France	SFPO
09CEB231		Fruit tart on pastry cream	NA	/	Vidas: positive	2009	France	SFPO
09CEB303	ERS4338887	unknown	NA	Merda et al, 2020	NA	1997	Japan	SFPO
09CEB314	ERS4338888	Cheese	NA	Merda et al, 2020	Vidas: positive	2009	France	SFPO
10CEB282		Steak	NA	/	Vidas: positive	2010	France	SFPO
10CEB401		Chicken	NA	/	Vidas: positive	2010	France	SFPO
11CEB110		Merguez	NA	/	Vidas: positive	2011	France	SFPO
12CEB137		Sheep's cheese	NA	/	Vidas: positive	2012	France	SFPO
12CEB368		Semi-cooked foie gras	NA	/	Vidas: positive	2012	France	SFPO
12CEB496		Chicken Sandwich	NA	/	Vidas: positive	2012	France	SFPO
12CEB512		Cottage cheese	NA	/	Ridascreen: negative	2012	France	SFPO
13CEB193	ERS4338917	Matured cheese	NA	Merda et al, 2020	Vidas: positive	2013	France	SFPO
13CEB437		Sausage Sandwich	NA	/	Ridascreen: negative	2013	France	SFPO
14A / FRI361	ERS4338950	Meat	NA	Merda et al, 2020	NA	1962	United Kingdom	Reference Strain
14SBCL1004		Ham	NA	/	Vidas: positive	2014	France	SFPO
14SBCL770		Pork stir-fry	NA	/	Ridascreen: negative	2014	France	SFPO

Strain	Accession no.	sample origin	Bacteria count (cfu/g)	Related publication	SE detected in food (official method)	Date of isolation	Geographical location	Strain origin
15SBCL1151		Cream puff	1.80E+08	/	Vidas: positive	2015	France	SFPO
15SBCL1428	ERS4338968	Cheese	6.40E+05	Merda et al, 2020	NA	2015	France	SFPO
15SBCL1438	ERS4338971	Cheese	1.00E+06	Merda et al, 2020	NA	2015	France	SFPO
15SBCL452		Paëlla	1.60E+06	/	Vidas: positive	2015	France	SFPO
16SBCL1028		Coriander Turkey	1.60E+03	/	No food sample	2016	France	SFPO
16SBCL1204		Chicken - rice	1.00E+04	/	Ridascreen: negative	2016	France	SFPO
16SBCL1368		Beef Bourguignon	10-100	/	No food sample	2016	France	SFPO
16SBCL246		Marinated salmon	1.10E+03	/	NA	2016	France	SFPO
16SBCL259		Kitchen tool	NA	/	No food sample	2016	France	SFPO
16SBCL899		Semolina couscous	NP	/	Vidas: positive	2016	France	SFPO
17SBCL25		Chicken	3.30E+05	/	Vidas: negative Ridascreen: negative	2017	France	SFPO
17SBCL330		vegetable	No food sample	/	NA	2017	France	SFPO
17SBCL693		Cream cake	1.50E+08	/	Vidas: positive	2017	France	SFPO
18SBCL601		Paella with meat	1.10E+09	/	Vidas: positive	2018	France	SFPO
18SBCL855		sandwich	< 400	/	Vidas: negative	2018	France	SFPO
19SBCL1059		Raw milk	1.00E+07	/	Vidas: positive	2019	France	Own Check
19SBCL591		Ham	1.40E+08	/	Vidas : positive	2019	France	SFPO
20SBCL08		Raw milk cheese	1.00E+06	/	Vidas : positive	2020	France	Own Check
337E		Pasteurized milk cheese	NA	Letertre et al 2003b	NA	unknown	unknown	SFPO
349E		ribs	NA	Letertre et al 2003a	NA	unknown	unknown	SFPO
352E		fresh cheese	NA	Letertre et al 2003a	NA	unknown	unknown	SFPO
356E		cheese	NA	Letertre et al 2003a	NA	unknown	unknown	SFPO
42A / FRIS6	ERS4339010	unknown	NA	Merda et al, 2020	NA	2001	unknown	Reference Strain
43A / FRI137	ERS4339011	Human	NA	Merda et al, 2020	NA	2001	unknown	Reference Strain
44A / FRI1230	ERS4339012	Breast milk	NA	Merda et al, 2020	NA	2001	unknown	Reference Strain
45A / FRI1151M	ERS4339013	unknown	NA	Merda et al, 2020	NA	2001	unknown	Reference Strain

Strain	Accession no.	sample origin	Bacteria count (cfu/g)	Related publication	SE detected in food (official method)	Date of isolation	Geographical location	Strain origin
46A / FRI326	ERS4339014	Chicken tetrazzini	NA	Merda et al, 2020	NA	2001	unknown	Reference Strain

NA: Not available. SFPO: Staphylococcal food poisoning outbreaks

Table S2. Peptides database of 24 SEs.

Toxin	Peptide	m/z	z	RT (min)	EC %	Fragments	Variant present	Variant absent	Commun SE	Peptide ratio	CV% ratio	Specific peptide	Heavy peptide
SEA	GLIVFHTSTEPSVNYDLFGAQGQYSNTLLR	1109.893	3+	14.9	18	y_9^+, y_{10}^+, y_{12}^+	2, 3	1, 4		0.67	18.28	x	
SEA	NVTVQELDLQAR	693.3728	2+	9.5	21	y_9^+, y_8^+, y_{10}^+	1, 3, 4	2		1.27	9.65	x	x
SEA	QNTVPLETVK	564.8166	2+	7.3	18	y_6^+, y_6^{++}, y_8^+	1, 2, 3, 4			1.00	0.00	x	x
SEA	SELQGTALGNLK	615.8381	2+	8.5	21	y_8^+, y_9^+, y_{10}^+	3, 4	1, 2		0.18	3.46		
SEA	YNLYNSDVFDGK	717.8304	2+	10	18	y_8^+, y_9^+, y_{10}^+	1, 3, 4	2		0.77	8.34	x	x
SEA	GFFTDHSWYNDLLVDFDSK	769.3515	3+	15.9	18	y_5^+, y_6^+, y_7^+	3	1, 2, 4		0.18	0.00		
SEA	QIYYYNEK	560.7691	2+	6	18	y_4^+, y_5^+, y_6^+	1, 3, 4	2	SEE1, 2, 3	0.48	17.08		
SEA	SELQGAALGNLK	600.8328	2+	8.5	18	y_6^+, y_8^+, y_9^+	1	2, 3, 4		0.25	0.00		
SEA	GLIVFHTSTEPSVNYDLFGAQGQNSNTLLR	1093.5529	3+	14.4	18	y_9^+, y_{10}^+, y_{12}^+	1, 4	2, 3		0.76	16.19		
SEA	GFFTNHSWYNDLLVDFDSK	769.0235	3+	15.4	18	y_5^+, y_6^+, y_7^+	1, 4	2, 3		0.04	120.85		
SEA	EVTVQELDLQAR	700.8726	2+	9.8	18	y_6^+, y_7^+, y_8^+	2	1, 3, 4	SEE1, 2, 3	0.61	0.00	x	
SEA	SELQGVALDNLR	657.8542	2+	10.1	18	y_5^+, y_6^+, y_8^+	2	1, 3, 4		0.86	0.00		
SEB	ESQPDPKPDELHK	507.2511	3+	3.2	15	y_6^+, y_8^{++}, y_{10}^{++}	1, 2, 3, 4			0.50	19.06		
SEB	FIENENSFWYDMMPAPGDK	764.331	3+	14.3	18	y_4^+, y_6^+, y_{12}^+	1	2, 3, 4		0.01	13.03		
SEB	FTGLMENMK	535.7541	2+	8.9	18	y_5^+, y_7^+, y_8^+	1, 3, 4	2	SEC 1, 2, 7, 8, 13	3.79	1.01		
SEB	IEVYLTTK	483.779	2+	8.4	18	y_5^+, y_6^+, y_7^+	1, 2, 3, 4			1.00	0.00	x	x
SEB	LGNYDNVR	475.7381	2+	4.1	21	y_4^+, y_6^+, y_7^+	1, 2, 3, 4			0.29	7.17		
SEB	NLLSFDVQTNK	639.8381	2+	10.7	15	y_7^+, y_8^+, y_9^+	1, 2, 3, 4			0.52	2.71		

Toxin	Peptide	m/z	z	RT (min)	EC %	Fragments	Variant present	Variant absent	Common SE	Peptide ratio	CV% ratio	Specific peptide	Heavy peptide
SEB	SIDQFLYFDLIYSIK	932.9902	2+	18.1	18	y_8^+, y_9^+, y_{10}^+	1, 2, 3, 4			0.43	17.40		
SEB	VLYDDNHVSAINVK	529.6106	3+	8.3	15	y_{11}^{++}, y_{12}^{++}, y_{13}^{++}	1, 2, 3, 4			4.04	10.63	x	x
SEB	VTAQELDYLTR	654.8433	2+	9.7	21	y_6^+, y_7^+, y_8^+	1, 2, 3, 4			1.53	1.04	x	x
SEB	YLMMYNDNK	596.2623	2+	8	18	y_5^+, y_6^+, y_7^+	1, 2, 3, 4		SEC 1, 2, 3, 4, 5, 7, 8, 10, 11, 13	0.92	0.72		
SEB	LYEFNNSPYETGYIK	919.4358	2+	10.8	21	y_8^+, y_9^+, y_{11}^+	1, 2, 3, 4			0.38	0.01		
SEB	FTVLMENMK	556.7776	2+	10.4	18	y_5^+, y_6^+, y_7^+	2	1, 3, 4		1.27	2.70		
SEC	ESQPDPTPDELHK	498.2353	3+	5.4	15	y_6^+, y_8^{++}, y_{10}^{++}	1, 2, 3, 4, 7, 8, 10, 11, 13	5		1.00	0.00	x	
SEC	FLAHDLIYNISDK	516.9401	3+	11.1	15	y_3^+, y_5^+, y_6^+	1, 2, 3, 4, 5, 7, 8, 10, 11, 13			0.09	13.67		
SEC	IEVHLTTK	314.1869	3+	6.2	18	y_5^{++}, y_6^{++}, y_7^{++}	1, 2, 3, 4, 5, 7, 8, 11, 13	10		0.40	10.80	x	
SEC	NLYEFNSSPYETGYIK	962.9518	2+	11.3	21	y_8^+, y_{11}^+, y_{12}^+	1, 2, 3, 4, 5, 7, 8, 10, 11, 13		SElU 1, 4, 5, 6, 9	0.35	7.03	x	x
SEC	NTISFEVQTDK	641.3197	2+	8.4	18	y_7^+, y_8^+, y_9^+	1, 2, 3, 4, 5, 7, 8, 10, 11, 13			0.04	1.20		
SEC	SSEFTGTMGNMK	430.5215	3+	7.2	18	y_2^+, y_4^+, y_5^+	3, 4, 5, 11	1, 2, 7, 8, 10, 13		0.01	15.25		
SEC	SVTAQELDIK	552.3008	2+	7.8	18	y_6^+, y_7^+, y_8^+	1, 2, 3, 4, 5, 7, 8, 10, 11, 13			0.96	3.01	x	
SEC	TELLNEDLAK	573.3061	2+	8.6	18	y_6^+, y_7^+, y_8^+	2, 3, 4, 5, 7, 8, 10, 11,	1		0.55	18.15		

Toxin	Peptide	m/z	z	RT (min)	EC %	Fragments	Variant present	Variant absent	Common SE	Peptide ratio	CV% ratio	Specific peptide	Heavy peptide
							13						
SEC	YLMMYNDNK	596.2623	2+	8	18	y_5^+, y_6^+, y_7^+	1, 2, 3, 4, 5, 7, 8, 10, 11, 13		SEB 1, 2, 3, 4	0.30	0.31		
SEC	YLYDDHYVSATK	492.2331	3+	7.3	15	y_9^{++}, y_{10}^{++}, y_{11}^{++}	3, 4, 5, 11	1, 2, 7, 8, 10, 13		1.31	0.04		
SEC	FTGLMENMK	535.7541	2+	8.9	18	y_5^+, y_7^+, y_8^+	1, 2, 7, 8, 13	3, 4, 5, 10, 11		0.61	4.72		
SEC	LQNVLIR	428.2742	2+	8	21	y_3^+, y_4^+, y_5^+	1, 2, 3, 4, 7, 8, 10, 11, 13	5	SEB 1, 3, 4	0.01	5.30		
SEC	VLYDDHYVSATK	470.9015	3+	6.9	18	y_9^{++}, y_{10}^{++}, y_{11}^{++}	1	2, 3, 4, 5, 7, 8, 10, 11, 13		1.14	1.68		
SEC	TELLNEGLAK	544.3033	2+	8.3	18	y_6^+, y_7^+, y_8^+	1	2, 3, 4, 5, 7, 8, 10, 11, 13		0.47	4.37		
SED	IPINLWINGVQK	697.9114	2+	13.2	21	y_7^+, y_9^+, y_{11}^{++}	1, 2, 3, 5			0.07	13.16		
SED	LYNNDTLGGK	547.7775	2+	5.2	18	y_7^+, y_8^+, y_9^+	1, 2, 3, 5			1.00	0.00	x	x
SED	NVDVYAIR	475.2587	2+	8	18	y_4^+, y_5^+, y_6^+	1, 2, 3, 5			0.69	1.51		
SED	NVTVQELDAQAR	672.3493	2+	7.7	21	y_7^+, y_8^+, y_{10}^+	1, 2, 3, 5			1.65	1.12	x	
SED	STGDQFLENTLLYK	814.912	2+	12.3	21	y_7^+, y_8^+, y_9^+	1, 2, 3, 5			0.67	3.12		
SED	VSYDLFDVK	543.2793	2+	11.3	18	y_6^+, y_7^+, y_8^+	1, 2, 3, 5			1.08	12.29	x	x
SED	NPIIGENK	442.7454	2+	4.2	18	y_4^+, y_5^+, y_7^{++}	1, 2, 3, 5			0.02	10.26		
SED	IEFDSSDESK	578.7539	2+	5.3	21	y_7^+, y_8^+, y_9^+	3	1, 2, 5		0.01	13.72		
SED	IEFDSSDGSK	542.7433	2+	5	18	y_7^+, y_8^+, y_9^+	1, 2, 5	3		0.06	11.79		
SEE	ESDDQFLENTLLFK	849.9147	2+	14.2	18	y_7^+, y_8^+, y_9^+	1, 2, 3			0.28	11.57		
SEE	EVTVQELDLQAR	700.8726	2+	9.8	21	y_6^+, y_7^+, y_8^+	1, 2, 3		SEA 2	1.30	13.38	x	x
SEE	FGLYNSDSFGGK	646.3013	2+	9.7	21	y_7^+, y_8^+,	1, 2, 3			1.00	0.00	x	x

Toxin	Peptide	m/z	z	RT (min)	EC %	Fragments	Variant present	Variant absent	Common SE	Peptide ratio	CV% ratio	Specific peptide	Heavy peptide
						y_9^+							
SEE	GFFTGHPWYNDLLVDLGSK	722.6933	3+	15.6	15	y_6^+, y_7^+, y_{17}^{++}	1	2, 3		1.73	18.59		
SEE	GLIVFHSSEGSTVSYDLFDAQGQYPDTLLR	1105.8771	3+	15.2	21	y_6^+, y_9^+, y_{12}^+	1, 3	2		0.72	0.36		
SEE	QIYYYNEK	560.7691	2+	6.1	18	y_4^+, y_5^+, y_6^+	1, 2, 3		SEA 1, 3, 4	1.16	13.32	x	
SEE	QTTVPIDK	451.2531	2+	5	21	y_2^+, y_4^+, y_6^+	1, 2, 3			1.17	15.40	x	x
SEE	SELQGIALSNLR	650.8646	2+	9.8	21	y_5^+, y_6^+, y_8^+	2	1, 3		0.05	11.77		
SEG	ELVPFVPYK	546.3104	2+	12.2	18	y_3^+, y_6^+, y_6^{++}	1, 2, 3, 4, 5, 7			2.30	16.41	x	
SEG	FLNIYGDNK	542.2771	2+	9.1	21	y_4^+, y_5^+, y_7^+	1, 2, 3, 4, 5, 7			0.65	13.29	x	x
SEG	NMVTIQELDYK	677.3396	2+	10.2	21	y_6^+, y_8^+, y_9^+	1, 2, 3, 4, 5, 7			1.21	3.78	x	
SEG	QFLSHDLIFPIEYK	583.9785	3+	13.7	18	y_5^+, y_5^{++}, y_6^+	1, 2, 3, 4, 5, 7			3.09	17.47	x	
SEG	QSLGFTITTNK	605.3273	2+	9.3	21	y_4^+, y_6^+, y_8^+	1, 2, 3, 4, 5, 7			1.00	0.00	x	x
SEG	GTMGNVMNLYTSPPVEGR	961.9586	2+	11.5	21	y_6^+, y_9^+, y_{12}^+	3, 7	1, 2, 4, 5		1.12	6.47		
SEG	LYEFDGSAFESGYIK	863.404	2+	12.3	18	y_{11}^+, y_{12}^+, y_{13}^+	1, 2, 3, 4, 5, 7			0.28	14.82		
SEH	VIGANVWVDGIQK	699.8906	2+	11.2	21	y_7^+, y_9^+, y_{11}^+	1, 2, 4	3		0.74	7.95		
SEH	DYSFDIYDLK	639.7981	2+	12.7	15	y_6^+, y_7^+, y_8^+	1, 2, 3, 4			0.18	2.25		
SEH	ETELIR	380.7136	2+	4.7	15	y_1^+, y_2^+, y_3^+	1, 2, 3, 4			0.45	9.22		
SEH	FATADLAQK	482.7585	2+	6.5	18	y_6^+, y_7^+, y_8^+	1, 2, 3, 4			0.66	3.43	x	x
SEH	GLIEFDMK	476.7441	2+	11	15	y_2^+, y_5^+, y_6^+	1, 2, 3, 4			0.77	15.76	x	
SEH	NVDIYGASFYYK	720.3457	2+	11	15	y_7^+, y_8^+, y_{10}^+	2, 3, 4	1		0.32	81.21		
SEH	SVDIYGASFYYK	706.8403	2+	11.1	18	y_7^+, y_8^+, y_{10}^+	1	2, 3, 4		0.25	46.56		
SEH	NVTLQELDIK	586.8297	2+	10.3	15	y_6^+, y_7^+, y_8^+	1, 2, 3, 4			0.71	27.18	x	x
SEH	SDDISHIDVNLYTK	540.6019	3+	9.8	15	y_{10}^{++}, y_{12}^{++}, y_{13}^{++}	1, 2, 3, 4			1.00	0.00	x	x
SEH	SELTDLALANAYGQYNHPFIK	789.0656	3+	13	18	y_{15}^{++}, y_{18}^{++}, y_{19}^{++}	1, 2, 3, 4			0.01	14.94		

Toxin	Peptide	m/z	z	RT (min)	EC %	Fragments	Variant present	Variant absent	Common SE	Peptide ratio	CV% ratio	Specific peptide	Heavy peptide
SEI	LVTAQEIDVK	558.319	2+	8	18	y_6^+, y_7^+, y_8^+	1, 2, 3, 4, 5, 6, 8, 10, 11, 12, 13, 15		SEK 5, 8	1.00	0.00	x	x
SEI	SFSYDLFYTGDGLPVSFLK	719.3577	3+	17.2	18	y_6^+, y_6^{++}, y_{10}^+	1, 3, 4, 5, 10	2, 6, 8, 11, 12, 13, 15		0.02	15.85		
SEI	VLFHLNNEK	371.8732	3+	7.6	15	y_6^{++}, y_7^{++}, y_8^{++}	3, 4, 15	1, 2, 5, 6, 8, 10, 11, 12, 13		2.98	19.46	x	
SEI	VLFHLNDEK	372.2012	3+	7.9	15	y_2^+, y_7^{++}, y_8^{++}	1, 2, 5, 6, 8, 10, 11, 12, 13	3, 4, 15		1.33	10.72	x	
SEI	YLQEEYNIYGHNNTGK	648.3025	3+	7.6	18	y_8^+, y_{14}^{++}, y_{15}^{++}	1, 3, 4, 6, 8, 11, 13, 15	2, 5, 10, 12		0.64	16.21	x	x
SEI	YLQEEYNIYGHNNNGK	653.9700	3+	7.4	15	y_{12}^{++}, y_{13}^{++}, y_{14}^{++}	5, 10	1, 2, 3, 4, 6, 8, 11, 12, 13, 15		0.38	8.06		
SEI	HDYIDLK	452.2322	2+	6.7	21	y_2^+, y_5^+, y_6^+	2, 3, 4, 6, 12, 15	1, 5, 8, 10, 11, 13		2.17	11.31		
SEI	QGDIGVGNLR	514.7778	2+	7.1	21	y_4^+, y_5^+, y_6^+	1, 2, 3, 4, 5, 6, 8, 10, 11, 12, 13, 15			0.73	13.24	x	x
SEI	IPINLWVNGK	579.3436	2+	11.6	21	y_7^+, y_8^+, y_9^{++}	1, 2, 3, 4, 6, 8, 11, 13, 15	5, 10, 12		0.25	11.75		
SEI	IPINLWINGK	586.3515	2+	12.4	21	y_7^+, y_8^+, y_9^{++}	5, 10	1, 2, 3, 4, 6, 8, 11, 12, 13, 15		0.28	12.69		
SElJ	SFFISHSQYNDLLVQFDSK	759.0392	3+	14	18	y_6^+, y_7^+, y_7^{++}	1, 2, 3, 4			0.04	25.71		
SElJ	IMTEDQFLDYTLLFK	626.3182	3+	16.5	18	y_6^+, y_7^+, y_8^+	1, 2, 3, 4			0.11	2.08	x	
SElJ	TVVPLDIVK	492.3104	2+	11.3	21	y_6^+, y_6^{++}, y_7^+	1, 2, 3, 4			0.01	35.15		

Toxin	Peptide	m/z	z	RT (min)	EC %	Fragments	Variant present	Variant absent	Common SE	Peptide ratio	CV% ratio	Specific peptide	Heavy peptide
SElJ	QYNLYNPSTFGGK	744.8595	2+	9.6	18	y_8^+, y_9^+, y_{11}^+	2, 3	1, 4		0.34	1.52	x	
SElJ	SELSSITLNNLR	673.8673	2+	10.8	18	y_4^+, y_6^+, y_9^+	1, 2, 3, 4			1.00	0.00	x	
SElJ	VTIQELDLQAR	643.3592	2+	10.1	18	y_6^+, y_7^+, y_8^+	1, 2, 3, 4			0.75	11.44	x	
SEK	DFVDLK	368.6974	2+	8.4	18	y_2^+, y_3^+, y_4^+	1, 3, 5, 7, 8	2, 4, 9		0.08	1.81		
SEK	DNDTPIANQLQFSNESYDLISESK	910.091	3+	13.4	18	y_8^+, y_{15}^{++}, y_{20}^{++}	1, 2, 3, 4, 5, 7, 8, 9			0.01	15.76		
SEK	FYSGFNIGK	516.7611	2+	9.5	18	y_6^+, y_7^+, y_8^+	1, 2, 3, 4, 5, 7, 8, 9			1.00	0.00	x	
SEK	NIPINIWINGNHK	766.9202	2+	11.6	21	y_7^+, y_{11}^+, y_{11}^{++}	1, 2, 3, 4, 5, 7, 8, 9			0.18	3.37	x	
SEK	QGDIGIDNLR	550.7884	2+	8.2	21	y_4^+, y_5^+, y_6^+	1, 2, 3, 4, 5, 7, 8, 9			0.81	7.52	x	
SEK	VTFHLNNNDTFSYDLFYTGDDGLPK	965.1154	3+	14.4	18	y_8^+, y_9^+, y_{10}^+	1, 2, 3, 4, 5, 7	8, 9		0.23	14.11		
SEK	YLQEEYNIYGHNGTK	610.2882	3+	7.6	18	y_7^+, y_{12}^{++}, y_{13}^{++}	1, 2, 3, 4, 5, 7, 8, 9			0.06	15.72		
SEK	FVTAQEIDVK	575.3111	2+	8.5	18	y_6^+, y_7^+, y_8^+	1, 4, 7	2, 3, 5, 8, 9		1.52	3.31		
SEK	LVTAQEIDVK	558.319	2+	8	18	y_6^+, y_7^+, y_8^+	5, 8	1, 2, 3, 4, 7, 9	SEI 1, 2, 3, 4, 5, 6, 8, 10, 11, 12, 13, 15, 17	1.22	5.14	x	
SEK	FVTAQEIDIK	582.319	2+	9.4	18	y_6^+, y_7^+, y_8^+	2, 9	1, 3, 4, 5, 7, 8		0.04	24.52		
SEL	FYSGFDK	432.2003	2+	6.7	21	y_2^+, y_5^+, y_6^+	1, 2, 4, 5, 6, 7, 8, 9, 10, 12, 13			0.51	1.51	x	
SEL	IIPINLIINGK	604.3923	2+	14	15	y_7^+, y_9^+, y_9^{++}	1, 2, 4, 5, 6, 7, 8, 9, 10, 12, 13			2.87	9.07	x	
SEL	MVTAQEIDVK	567.2972	2+	7.7	18	y_6^+, y_7^+, y_8^+	1, 2, 4, 5, 6, 7,			1.12	12.32		

Toxin	Peptide	m/z	z	RT (min)	EC %	Fragments	Variant present	Variant absent	Commun SE	Peptide ratio	CV% ratio	Specific peptide	Heavy peptide
							8, 9, 10, 12, 13						
SEL	NVDVFGISYK	571.298	2+	11.1	15	y_6^+, y_7^+, y_8^+	1, 2, 4, 5, 6, 7, 8, 9, 10, 12, 13			1.36	15.18	x	
SEL	YEYVNLK	464.7424	2+	7.4	18	y_4^+, y_5^+, y_6^+	1, 2, 4, 5, 6, 7, 8, 9, 12, 13	10		1.00	0.00	x	
SEL	YLQDEFNIYGHNDTGK	638.627	3+	9.4	15	y_8^+, y_{13}^{++}, y_{14}^{++}	1, 2, 4, 5, 6, 7, 8, 9, 10, 12, 13			0.50	18.91		
SEL	TIYGGVTK	419.7371	2+	4.7	15	y_5^+, y_6^+, y_7^+	1, 2, 4, 5, 6, 7, 8, 9, 10, 12, 13			0.89	1.74		
SEL	NVDIFGISYK	578.3059	2+	12.1	18	y_5^+, y_6^+, y_8^+	8, 12	1, 2, 4, 5, 6, 7, 9, 10, 13		1.11	5.34		
SEM	DVGVLNLR	443.2613	2+	9.8	18	y_3^+, y_4^+, y_6^+	1, 2, 3, 4, 5, 6, 8, 9, 15			1.00	0.00	x	x
SEM	ILFHLNDGSSFSYDLFDTGTGQAESFLK	1037.1645	3+	16.2	18	y_8^+, y_{12}^+, y_{13}^+	3, 4, 8, 15	1, 2, 5, 6, 9		0.13	18.46		
SEM	LVTAQEIDTK	559.3086	2+	6.5	18	y_6^+, y_7^+, y_8^+	3, 4, 6, 8, 15	1, 2, 5, 9		0.68	2.98	x	
SEM	IVTAQEIDTK	559.3086	2+	6.3	21	y_6^+, y_7^+, y_8^+	1, 2, 5, 9	3, 4, 6, 8, 15		0.61	0.22	x	
SEM	YLQEEYNIYGFNDTNK	1005.9576	2+	10.8	21	y_7^+, y_8^+, y_{12}^+	1, 2, 3, 4, 5, 6, 8, 9, 15			0.10	4.11	x	
SEM	YIYGGVTLAGDYLEK	831.4247	2+	12.2	18	y_9^+, y_{12}^+, y_{13}^+	3, 4, 6, 8, 15	1, 2, 5, 9		0.19	13.32		
SEM	YMYGGVTLAGDYLEK	840.4029	2+	11.9	18	y_9^+, y_{12}^+, y_{13}^+	1, 2, 5, 9	3, 4, 6, 8, 15		0.02	23.85		
SEN	DGVQQEGFVIK	610.3195	2+	9	18	y_6^+, y_7^+, y_8^+	1, 2, 3, 5, 6, 7, 8	4, 9		0.42	8.70		
SEN	GSVGAEFFQFYSDNR	862.3892	2+	12.8	21	y_8^+, y_9^+, y_{10}^+	1, 2, 3, 4, 5, 6, 7, 8	9		0.97	3.94		
SEN	ISTDQLLNNTIILK	793.4616	2+	13.3	18	y_7^+, y_8^+,	1, 2, 3,	9		0.15	25.87		

Toxin	Peptide	m/z	z	RT (min)	EC %	Fragments	Variant present	Variant absent	Common SE	Peptide ratio	CV% ratio	Specific peptide	Heavy peptide
						y_9^+	4, 5, 6, 7, 8						
SEN	NIDISVLK	451.2713	2+	10.2	15	y_4^+, y_5^+, y_6^+	1, 2, 3, 5, 6, 7, 8	4, 9		1.92	3.79	x	
SEN	NIDIYGLYFGNK	708.8615	2+	13.2	18	y_7^+, y_8^+, y_{10}^+	3, 5, 7	1, 2, 4, 6, 8, 9		1.44	20.30	x	
SEN	VTVQELDTK	516.7822	2+	6.4	18	y_6^+, y_7^+, y_8^+	1, 2, 3, 4, 5, 6, 7, 8, 9			1.00	0.00	x	x
SEO	FLDFDLLFK	579.3157	2+	16.7	18	y_6^+, y_7^+, y_8^+	1, 2, 3, 5, 6, 7, 8, 9, 14, 19, 21	4		0.44	128.25	x	
SEO	GNLPDQYLQIYNDNK	598.9601	3+	11.05	18	y_5^+, y_8^+, y_{12}^{++}	1, 2, 3, 4, 6, 7, 9, 14, 19, 21	5, 8		0.08	3.52		
SEO	SSVDPIALHNINDDYINNR	724.0223	3+	10.4	21	y_5^+, y_8^+, y_{15}^{++}	3, 6, 8, 21	1, 2, 4, 5, 7, 9, 14, 19		0.59	2.71		
SEO	TVDIYGVYYK	610.8135	2+	10	18	y_6^+, y_7^+, y_8^+	1, 2, 3, 4, 5, 6, 7, 8, 9, 14, 19, 21			1.00	0.00	x	
SEO	VTAQELDIK	508.7848	2+	7.3	18	y_6^+, y_8^+, y_8^{++}	1, 2, 3, 4, 5, 6, 7, 8, 9, 14, 19, 21			0.42	42.96	x	x
SEO	SINWLDGISAK	602.322	2+	11.8	18	y_7^+, y_8^+, y_9^+	6, 21	1, 2, 3, 4, 5, 7, 8, 9, 14, 19		0.18	99.54		
SEO	SINWLDGISAEFK	740.3775	2+	14.7	21	y_8^+, y_9^+, y_{11}^+	3, 8, 14	1, 2, 4, 5, 6, 7, 9, 19, 21		0.22	90.40		
SEO	ESFYYDLFYIK	744.3583	2+	14.8	18	y_6^+, y_7^+, y_8^+	1, 2, 3, 4, 5, 6, 7, 9, 14, 19, 21	8		0.03	141.42		
SEP	ESNDQFLK	490.7378	2+	5.4	18	y_2^+, y_3^+, y_6^+	2, 3, 4			0.17	0.13		

Toxin	Peptide	m/z	z	RT (min)	EC %	Fragments	Variant present	Variant absent	Commun SE	Peptide ratio	CV% ratio	Specific peptide	Heavy peptide
SEP	EVTVQELDLQSR	708.8701	2+	9.4	21	y_{6}^{+}, y_{7}^{+}, y_{8}^{+}	2, 3	4		0.89	4.40	x	
SEP	GLIEFHPSSGDSVGYDLFGAQGQYPDTQLR	1085.5178	3+	14	18	y_{6}^{+}, y_{9}^{+}, y_{12}^{+}	2, 3, 4			1.12	0.55	x	
SEP	QNTVPLGTVK	528.806	2+	7	21	y_{6}^{+}, y_{7}^{+}, y_{8}^{+}	2, 3, 4			1.00	0.00	x	
SEP	SELQGTALSNLR	644.8464	2+	8.7	18	y_{4}^{+}, y_{8}^{+}, y_{9}^{+}	2, 3, 4		SEE 3	0.45	7.47	x	x
SEQ	DVGVINLR	443.2613	2+	9.3	15	y_{3}^{+}, y_{4}^{+}, y_{6}^{+}	1, 2, 3, 4, 5			2.08	2.74	x	
SEQ	EVTAQEIDIK	573.3061	2+	7.7	18	y_{5}^{+}, y_{6}^{+}, y_{8}^{+}	1, 2, 3, 4, 5			1.00	0.00	x	x
SEQ	FNSGFNK	407.1981	2+	3.6	18	y_{2}^{+}, y_{5}^{+}, y_{6}^{+}	1, 2, 3, 4, 5			0.30	6.16		
SEQ	ITFHLNNETSFTYDLFYTGTGQAESFLK	1082.1873	3+	16.2	18	y_{10}^{+}, y_{11}^{+}, y_{20}^{++}	5	1, 2, 3, 4		0.03	16.10		
SEQ	ITFHLNNEPSFTYDLFYTGTGQAESFLK	1080.8557	3+	16.2	18	y_{10}^{+}, y_{11}^{+}, y_{12}^{+}	1, 2, 3, 4	5		0.13	18.50		
SEQ	NFYTNYQPETLQGVSSGNFSTSHQLEYIDGK	881.9115	4+	12.2	18	y_{17}^{++}, y_{19}^{++}, y_{20}^{++}	5	1, 2, 3, 4		0.10	0.87		
SEQ	NIPINLWVNGK	634.3615	2+	12.5	15	y_{7}^{+}, y_{9}^{+}, y_{9}^{++}	1, 2, 3, 4, 5			0.94	7.98	x	
SEQ	QNTISTDEVSTQK	725.8546	2+	5.2	21	y_{8}^{+}, y_{9}^{+}, y_{11}^{+}	5	1, 2, 3, 4		0.05	0.04		
SEQ	YLQNEYNIYGFNK	833.399	2+	10.4	21	y_{5}^{+}, y_{8}^{+}, y_{10}^{+}	1, 2, 3, 4, 5			0.33	3.47		
SEQ	YMYGGITLANQNLDKPR	651.9999	3+	9.9	15	y_{11}^{++}, y_{14}^{++}, y_{15}^{++}	1, 2, 3, 4, 5			1.50	5.12		
SEQ	YTLYSQFHNEYEAK	896.9125	2+	8.6	21	y_{8}^{+}, y_{9}^{+}, y_{10}^{+}	1, 2, 3, 4, 5			0.02	2.22		
SEQ	LQGVSSGNFSTSHQLEYIDGK	756.3695	3+	9.6	18	y_{16}^{++}, y_{17}^{++}, y_{19}^{++}	1, 2, 3, 4	5		0.99	1.24		
SEQ	NFYANYQPEK	637.296	2+	7.2	18	y_{3}^{+}, y_{7}^{+}, y_{8}^{+}	2	1, 3, 4, 5		0.65	1.94		
SER	DLVPFIPYK	546.3104	2+	13.6	18	y_{3}^{+}, y_{6}^{+}, y_{6}^{++}	1, 2, 4, 5			2.64	4.10		
SER	FVNIYGDNK	535.2693	2+	7.7	18	y_{4}^{+}, y_{5}^{+}, y_{7}^{+}	1, 2, 4, 5			5.16	1.96	x	x
SER	GTMGNVESLYK	599.7923	2+	8.3	18	y_{5}^{+}, y_{8}^{+}, y_{9}^{+}	1, 2, 4, 5			0.02	141.42		
SER	NIIVPVTVDNK	606.3533	2+	10.1	15	y_{7}^{+}, y_{8}^{+}, y_{9}^{+}	1, 2, 4, 5			3.84	8.19		
SER	QFLGHDLIFPIPYSEYK	689.691	3+	14.47	18	y_{6}^{+}, y_{6}^{++}, y_{8}^{+}	1, 2, 5	4		2.43	48.26		
SER	TVTVQELDYK	598.3139	2+	8.1	18	y_{6}^{+}, y_{7}^{+},	1, 2, 4,			2.33	9.17	x	x

Toxin	Peptide	m/z	z	RT (min)	EC %	Fragments	Variant present	Variant absent	Common SE	Peptide ratio	CV% ratio	Specific peptide	Heavy peptide
						y_8^+	5						
SER	QQFSFTISTNK	650.8302	2+	9.7	18	y_4^+, y_6^+, y_9^+	1, 2, 4, 5			0.08	20.94		
SER	SEFINK	369.1951	2+	3.3	18	y_2^+, y_3^+, y_4^+	1, 2, 4, 5			1.00	0.00		
SER	TIDASSVK	410.7242	2+	2.8	15	y_5^+, y_6^+, y_7^{++}	1, 2, 4, 5			0.80	27.23		
SER	AVIAENVK	422.2504	2+	4.8	15	y_4^+, y_5^+, y_6^+	1, 2, 4, 5			9.45	3.47	x	
SER	NWLTNNK	445.23	2+	5.9	18	y_4^+, y_5^+, y_6^+	1, 2, 4, 5			0.44	2.73		
SES	EPTIQELDLK	593.3217	2+	9.8	18	y_3^+, y_5^+, y_6^+	1, 3			0.79	16.79		
SES	GFMITTDK	456.7284	2+	7.9	18	y_4^+, y_5^+, y_6^+	1, 3			0.31	11.35		
SES	NSIGVNVFK	489.2744	2+	9.1	18	y_2^+, y_6^+, y_7^+	1, 3			6.92	0.19	x	
SES	SELDSTALYNIK	677.3485	2+	9.9	18	y_8^+, y_9^+, y_{10}^+	1, 3			1.00	0.00	x	x
SES	TNSTQLLSNDLIFDDITLK	717.7108	3+	15.5	18	y_6^+, y_7^+, y_8^+	1, 3			1.00	0.05		
SES	TSYSQDNIILDIK	755.3934	2+	11.3	21	y_5^+, y_8^+, y_{10}^+	1, 3			4.70	4.69	x	
SES	YSVDFLK	436.2316	2+	9.9	18	y_4^+, y_5^+, y_6^+	1, 3			5.08	2.35	x	
SET	EGLKDFYSK	362.8537	3+	7	15	y_1^+, y_2^+, y_3^+	1			0.82	NR		
SET	FYDGDISK	472.7216	2+	6	18	y_5^+, y_6^+, y_7^+	1			1.00	NR	x	
SET	INTLDDKILNQFK	521.291	3+	10.9	15	y_9^{++}, y_{11}^{++}, y_{12}^{++}	1			0.63	NR		
SET	ISHFDIYMEK	428.2094	3+	9	15	y_3^+, y_4^+, y_9^{++}	1			0.44	NR		
SET	IDVYTNK	426.7267	2+	4.4	15	y_4^+, y_5^+, y_6^+	1			0.05	NR		
SET	ELEIGNR	415.722	2+	5.1	18	y_3^+, y_4^+, y_6^{++}	1			0.06	NR		
SET	INTLDDK	409.7163	2+	2.5	18	y_2^+, y_5^+, y_6^+	1			0.25	NR		
SET	HSPLDKPTNISYR	509.9353	3+	6.7	18	y_5^+, y_{11}^{++}, y_{12}^{++}	1			0.55	NR	x	
SET	VGDYVDAWGHIINNKPIGK	699.369	3+	11.4	18	y_{14}^{++}, y_{15}^{++}, y_{18}^{++}	1			0.63	NR	x	
SElU	ASEFTGLMDNMR	686.3052	2+	10.6	18	y_5^+, y_7^+, y_8^+	1, 2, 4, 5, 6, 9,	7, 17		0.93	0.84	x	

Toxin	Peptide	m/z	z	RT (min)	EC %	Fragments	Variant present	Variant absent	Common SE	Peptide ratio	CV% ratio	Specific peptide	Heavy peptide
							10, 11						
SElU	FLQHDLLFK	387.5536	3+	10.3	18	y_1^+, y_2^+, y_8^+	1, 2, 4, 5, 7, 9, 10, 11, 17	6		0.04	9.01	x	
SElU	NITAQEIDYK	597.8037	2+	7.3	18	y_6^+, y_7^+, y_8^+	1, 2, 4, 5, 6, 7, 9, 10, 11, 17			1.03	2.59	x	
SElU	YLLIYNDNK	578.3059	2+	9.4	18	y_5^+, y_6^+, y_7^+	1, 2, 4, 5, 6, 7, 9, 10, 11, 17			1.00	0.00	x	
SEX	GNIVINTK	429.7558	2+	5.4	18	y_4^+, y_5^+, y_6^+	1, 3, 4, 5, 7, 9, 10, 12, 14, 15, 16, 17, 22, 24, 25, 28, 34, 36, 41, 45			4.69	3.11	x	
SEX	NVTFELVK	475.2713	2+	9.8	18	y_2^+, y_5^+, y_6^+	1, 4, 7, 12, 14, 17, 22, 24, 25,28, 34, 36, 41	3, 5, 9, 10, 15, 16, 45		2.33	3.93	x	
SEX	INLEGTYR	483.2562	2+	7	18	y_4^+, y_5^+, y_6^+	3, 10, 16	1, 4, 5, 7, 9, 12, 14, 15, 17, 22, 24, 25, 28, 34, 36, 41, 45		7.38	7.15		
SEX	IHLEGTYTVAGR	439.5701	3+	7.6	18	y_3^+, y_4^+, y_5^+	1, 4, 5, 15, 24, 25, 28, 34, 45	3, 7, 9, 10, 12, 14, 16, 17, 22, 36, 41		2.51	15.36		
SEX	EVVTLK	344.7156	2+	5.2	18	y_2^+, y_3^+, y_4^+	1, 3, 4, 5, 7, 9, 10, 12,	17, 41		2.68	1.86		

Toxin	Peptide	m/z	z	RT (min)	EC %	Fragments	Variant present	Variant absent	Common SE	Peptide ratio	CV% ratio	Specific peptide	Heavy peptide
							14, 15, 16, 22, 24, 25, 28, 34, 36, 45						
SEX	NWVYSERPLNENQVR	635.3185	3+	8.5	18	y_{11}^{++}, y_{12}^{++}, y_{13}^{++}	1, 3, 4, 5, 7, 9, 12, 14, 16, 17, 22, 24, 25 28, 36, 41, 45	10, 34		5.49	2.57	x	
SEX	ELDHIIR	299.1714	3+	6.4	18	y_{3}^{+}, y_{4}^{++}, y_{5}^{++}	1, 3, 4, 5, 7, 9, 10, 12, 14, 15, 16, 17, 22, 24, 25, 34, 36, 41, 45	28		3.27	2.73	x	
SEX	INTADIK	387.7214	2+	3.6	18	y_{2}^{+}, y_{5}^{+}, y_{6}^{+}	3, 5, 9, 10, 28, 45	1, 4, 7, 12, 14, 15, 16, 17, 22, 24, 25, 34, 36, 41		4.40	9.03		
SEX	NITLNK	351.7109	2+	3.3	18	y_{2}^{+}, y_{3}^{+}, y_{4}^{+}	1, 3, 4, 5, 7, 9, 10, 12, 14, 15, 16, 17, 22, 24, 25, 28, 34, 36, 41, 45			1.00	0.00		
SEY	TVLYNTDYLK	615.3243	2+	9.4	18	y_{6}^{+}, y_{7}^{+}, y_{8}^{+}	1, 2, 3, 4, 6, 7			3.17	4.99	x	
SEY	VNPDSLLEVK	557.3111	2+	10.1	18	y_{8}^{+}, y_{8}^{++}, y_{9}^{++}	1, 2, 3, 4, 6, 7			5.00	2.03	x	
SEY	EVTGVGENYIDVK	711.8592	2+	9.1	18	y_{7}^{+}, y_{8}^{+}, y_{10}^{+}	6	1, 2, 3, 4, 7		0.99	1.44		
SEY	YDPISK	361.6896	2+	3.4	21	y_{3}^{+}, y_{4}^{+},	1, 2, 3,			1.08	0.29		

Toxin	Peptide	m/z	z	RT (min)	EC %	Fragments	Variant present	Variant absent	Commun SE	Peptide ratio	CV% ratio	Specific peptide	Heavy peptide
						y_4^{++}	4, 6, 7						
SEY	VDTYNVR	433.722	2+	3.3	21	y_3^+, y_5^+, y_6^+	1, 2, 3, 4, 6, 7			1.00	0.00	x	
SEY	MNLFVNGHQTK	430.2204	3+	7.2	18	y_7^{++}, y_8^{++}, y_9^{++}	1, 2, 3, 4, 6, 7			0.15	3.79		
SEY	TFDFDNISHIDIYMK	620.2942	3+	13.8	18	y_{12}^{++}, y_{13}^{++}, y_{14}^{++}	1, 2, 3, 4, 6, 7			1.43	19.21		
SEY	VTNLSYR	426.7323	2+	4.3	18	y_3^+, y_5^+, y_6^+	1, 2, 3, 4, 6, 7			1.28	2.16	x	
SEZ	SSQYTGSWHNIWYLYNSDPVNAK	910.7576	3+	12.9	18	y_5^+, y_8^+, y_9^+	1, 2, 3, 5			0.41	1.35	x	
SEZ	YLMIYR	429.7307	2+	9.1	15	y_3^+, y_4^+, y_5^+	1, 2, 3, 5			0.77	3.55	x	
SEZ	IQPIVVK	398.7682	2+	7.5	15	y_2^+, y_3^+, y_4^+	1, 2, 3, 5			1.00	0.00	x	
SEZ	INLYHLGGTSYETGYIK	643.6633	3+	10.9	18	y_6^+, y_{15+}^+, y_{16+}^+	1, 2, 3, 5			0.36	0.80	x	
SEZ	YYWYDMMPDPGFTQSK	1014.929	2+	14.1	18	y_7^+, y_9^+, y_{10}^+	1, 2, 3, 5			0.25	11.57	x	
TSST 1	HQLTQIHGLYR	455.9177	3+	8	18	y_4^+, y_5^+, y_9^{++}	1, 3, 5, 6	2, 4		0.16	6.05		
TSST 1	LPTPIELPLK	560.8524	2+	13	21	y_7^+, y_7^{++}, y_9^{++}	1, 2, 3, 4, 5, 6			0.79	18.63	x	
TSST 1	ITMNDGSTYQSDLSK	830.3802	2+	7.6	21	y_{10}^+, y_{11}^+, y_{12}^+	1, 2, 3, 4, 5, 6			1.00	0.00	x	
TSST 1	NTDGSISLIIFPSPYYSPAFTK	1209.615	2+	16.5	18	y_5^+, y_{11}^+, y_{12}^+	1, 3, 6	2, 4, 5		1.08	6.79		
TSST 1	QLAISTLDFEIR	703.3879	2+	13.7	18	y_8^+, y_9^+, y_{10}^+	1, 3, 5, 6	2, 4		4.75	10.82	x	
TSST 1	QLAISTLDFK	568.3215	2+	11.6	18	y_5^+, y_6^+, y_7^+	2, 4	1, 3, 5, 6		1.71	4.72	x	
TSST 1	DLLDWYSSGSDAFTNSEVLDNSLGSMR	993.7819	3+	16.6	18	y_8^+, y_9^+, y_{10}^+	2, 4, 5	1, 3, 6		0.02	8.31		
TSST 1	FEYNTEKPPINIDEIK	650.6667	3+	10.96	18	y_9^+, y_{13}^{++}, y_{14}^{++}	1, 2, 3, 4, 5, 6			0.15	6.51		
TSST 1	HQLTQTHGLYR	451.9056	3+	4.2	18	y_4^+, y_9^{++}, y_{10}^{++}	2, 4	1, 3, 5, 6		0.22	8.13		

Figure S1. Sequence alignment of 24 SE and 164 variants.

SEA

```
a_2        MKKRKTAFILTLCIVIMLVTSPLVNGSEKSEEIKEKDLHKKSELQGVALDNLRQIYYHNG     60
a_3        --MKKTAFTLLLFIALTLTTSPLVNGSEKSEEINEKDLRKKSELQGTALGNLKQIYYYNE     58
a_1        --MKKTAFILLLFIALTWTTSPLVNGSEKSEEINEKDLRKKSELQGAALGNLKQIYYYNE     58
a_4        --MKKTAFILLLFIALTWTTSPLVNGSEKSEEINEKDLRKKSELQGTALGNLKQIYYYNE     58
              :**** * * *.:  .**************:****:*******.**.**:****:*

a_2        KAITENKESDNQFLQHTILFNGFFTDHPWYNDLLVDFDSKVLADKYKGKKVDLYGAYYGY     120
a_3        KAKTENKESHDQFLQHTILFKGFFTDHSWYNDLLVDFDSKDIVDKYKGKKVDLYGAYYGY     118
a_1        KAKTENKESHDQFLQHTILFKGFFTNHSWYNDLLVDFDSKDIVDKYKGKKVDLYGAYYGY     118
a_4        KAKTENKESHDQFLQHTILFKGFFTNHSWYNDLLVDFDSKDIVDKYKGKKVDLYGAYYGY     118
           ** ******.:*********:****:* ************ :.*****************

a_2        QCAGGTPNKTACMYGGVTLHDNNRLTEEKKVPINLWLDGKQNTVPLETVKTNKKEVTVQE     180
a_3        QCAGGTPNKTACMYGGVTLHDNNRLTEEKKVPINLWLDGKQNTVPLETVKTNKKNVTVQE     178
a_1        QCAGGTPNKTACMYGGVTLHDNNRLTEEKKVPINLWLDGKQNTVPLETVKTNKKNVTVQE     178
a_4        QCAGGTPNKTACMYGGVTLHDNNRLTEEKKVPINLWLDGKQNTVPLETVKTNKKNVTVQE     178
           ******************************************************:*****

a_2        LDLQARHYLHGKYNLYNSDTFDGKVQRGLIVFHTSTEPSVNYDLFGAQGQYSNTLLRIYR     240
a_3        LDLQARRYLQEKYNLYNSDVFDGKVQRGLIVFHTSTEPSVNYDLFGAQGQYSNTLLRIYR     238
a_1        LDLQARRYLQEKYNLYNSDVFDGKVQRGLIVFHTSTEPSVNYDLFGAQGQNSNTLLRIYR     238
a_4        LDLQARRYLQEKYNLYNSDVFDGKVQRGLIVFHTSTEPSVNYDLFGAQGQNSNTLLRIYR     238
           ******:**: ********.****************************** *********

a_2        DNKTINSENMHIDIYLYTT      259
a_3        DNKTINSENMHIDIYLYTS      257
a_1        DNKTINSENMHIDIYLYTS      257
a_4        DNKTINSENMHIDIYLYTS      257
           ******************:
```

SEB

```
b_3        MYNRLFVSRVILIFALILVIYTPNVLAESQPDPKPDELHKASKFTGLMENMKVLYDDNHV     60
b_1        MYKRLFISHVILIFALILVISTPNVLAESQPDPKPDELHKSSKFTGLMENMKVLYDDNHV     60
b_2        MYKRLFISHVILIFVLILVISTPNVLAESQPDPKPDELHKASKFTVLMENMKVLYDDNHV     60
b_4        MYKRLFISHVILIFVLILVISTPNVLAESQPDPKPDELHKASKFTGLMENMKVLYDDNHV     60
           **:***:*:*****.***** *******************:**** **************

b_3        SAINVKSIDQFLYFDLIYSIKDTKLGNYDNVRVEFKNKDLADKYKDKYVDVFGANYYYQC     120
b_1        SAINVKSIDQFLYFDLIYSIKDTKLGNYDNVRVEFKNKDLADKYKDKYVDVFGANYYYQC     120
b_2        SAINVKSIDQFLYFDLIYSIKDTKLGNYDNVRVEFKNKDLADKYKDKYVDVFGANYYYQC     120
b_4        SAINVKSIDQFLYFDLIYSIKDTKLGNYDNVRVEFKNKDLADKYKDKYVDVFGANYYYQC     120
           ************************************************************

b_3        YFSKKTNDINSHQTDKRKTCMYGGVTEHNGNHLDKYRSITVRVFEDGKNLLSFDVQTNKK     180
b_1        YFSKKTNDINSHQTDKRKTCMYGGVTEHNGNQLDKYRSITVRVFEDGKNLLSFDVQTNKK     180
b_2        YFSKKTNDINSHQTDKRKTCMYGGVTEHNGNQLDKYRSITVRVFEDGKNLLSFDVQTNKK     180
b_4        YFSKKTNDINSHQTDKRKTCMYGGVTEHNGNQLDKYRSITVRVFEDGKNLLSFDVQTNKK     180
           *******************************:****************************

b_3        KVTAQELDYLTRHYLVKNKKLYEFNNSPYETGYIKFIESENSFWYDMMPAPGDKFDQSKY     240
b_1        KVTAQELDYLTRHYLVKNKKLYEFNNSPYETGYIKFIENENSFWYDMMPAPGDKFDQSKY     240
b_2        KVTAQELDYLTRHYLVKNKKLYEFNNSPYETGYIKFIESENSFWYDMMPAPGDKFDQSKY     240
b_4        KVTAQELDYLTRHYLVKNKKLYEFNNSPYETGYIKFIESENSFWYDMMPAPGDKFDQSKY     240
           **************************************.*********************

b_3        LMMYNDNKLVDSKDVKIEVYLTTKKK       266
b_1        LMMYNDNKMVDSKDVKIEVYLTTKKK       266
b_2        LMMYNDNKLVDSKDVKIEVYLTTKKK       266
b_4        LMMYNDNKLVDSKDVKIEVYLTTKKK       266
```

```
        ********:*****************
```

SEC

```
c_10      MNKSRFISCVILIFALILVLFTPNVLAESQPDPTPDELHKSSEFTGTMGNMKYLYDDHYV      60
c_5       MYKRLFISRVILIFALILVISTPNVLAESQPDPMPDDLHKSSEFTGTMGNMKYLYDDHYV      60
c_11      MNKSRFISCVILIFALILVLFTPNVLAESQPDPTPDELHKSSEFTGTMGNMKYLYDDHYV      60
c_3       MNKSRFISCVILIFALILVLFTPNVLAESQPDPTPDELHKSSEFTGTMGNMKYLYDDHYV      60
c_4       MNKSRFISCVILIFALILVLFTPNVLAESQPDPTPDELHKSSEFTGTMGNMKYLYDDHYV      60
c_2       MNKSRFISCVILIFSLILVLFTPNVLAESQPDPTPDELHKASKFTGLMENMKVLYDDRYV      60
c_1       MNKSRFISCVILIFALILVLFTPNVLAESQPDPTPDELHKASKFTGLMENMKVLYDDHYV      60
c_13      MNKSRFISCVILIFALILVLFTPNVLAESQPDPTPDELHKASKFTGLMENMKVLYDDRYV      60
c_7       MNKSRFISCVILIFALILVLFTPNVLAESQPDPTPDELHKASKFTGLMENMKVLYDDRYV      60
c_8       MNKSRFISCVILIFALILVLFTPNVLAESQPDPTPDELHKASKFTGLMENMKVLYDDRYV      60
          * *  *** *****:****: *********** **:***:*:*** * *** ****:**

c_10      SATKVMSVDKFLAHDLIYNISDKKLKNYDIVKTELLNEDLAKKYKDEVVDVYGSNYYVNC      120
c_5       SATKVKSVDKFLAHDLIYNISDKKLKNYDKVKTELLNEDLAKKYKDEVVDVYGSNYYVNC      120
c_11      SATKVKSVDKFLAHDLIYNISDKKLKNYDKVKTELLNEDLAKKYKDEVVDVYGSNYYVNC      120
c_3       SATKVMSVDKFLAHDLIYNISDKKLKNYDKVKTELLNEDLAKKYKDEVVDVYGSNYYVNC      120
c_4       SATKVMSVDKFLAHDLIYNISDKKLKNYDIVKTELLNEDLAKKYKDEVVDVYGSNYYVNC      120
c_2       SATKVKSVDKFLAHDLIYNISDKKLKNYDKVKTELLNEDLAKKYKDEVVDVYGSNYYVNC      120
c_1       SATKVKSVDKFLAHDLIYNISDKKLKNYDKVKTELLNEGLAKKYKDEVVDVYGSNYYVNC      120
c_13      SATKVKSVDKFLAHDLIYNISDKKLKNYDKVKTELLNEDLAKKYKDEVVDVYGSNYYVNC      120
c_7       SATKVKSVDKFLAHDLIYNISDKKLKNYDKVKTELLNEDLAKKYKDEVVDVYGSNYYVNC      120
c_8       SATKVKSVDKFLAHDLIYNISDKKLKNYDKVKTELLNEDLAKKYKDEVVDVYGSNYYVNC      120
          ***** *********************** ******** .********************

c_10      YFSSKDNVGKVTGGKTCMYGGITKHEGNHFDNGNLQNVLIRVYENKRNTISFEVQTDKKS      180
c_5       YFSSKDNVGKVTGGKTCMYGGITKHEGNHFDNGNLQNVLVRVYENKRNTISFEVQTDKKS      180
c_11      YFSSKDNVGKVTGGKTCMYGGITKHEGNHFDNGNLQNVLIRVYENKRNTISFEVQTDKKS      180
c_3       YFSSKDNVGKVTGGKTCMYGGITKHEGNHFDNGNLQNVLIRVYENKRNTISFEVQTDKKS      180
c_4       YFSSKDNVGKVTGGKTCMYGGITKHEGNHFDNGNLQNVLIRVYENKRNTISFEVQTDKKS      180
c_2       CFSSKDNVGKVTGGKTCMYGGITKHEGNHFDNGNLQNVLIRVYENKRNTISFEVQTDKKS      180
c_1       YFSSKDNVGKVTGGKTCMYGGITKHEGNHFDNGNLQNVLIRVYENKRNTISFEVQTDKKS      180
c_13      YFSSKDNVGKVTGGKTCMYGGITKHEGNHFDNGKLQNVLIRVYENKRNTISFEVQTDKKS      180
c_7       YFSSKDNVGKVTGGKTCMYGGITKHEGNHFDNGKLQNVLIRVYENKRNTISFEVQTDKKS      180
c_8       YFSSKDNVGKVTGGKTCMYGGITKHEGNHFDNGNLQNVLIRVYENKRNTISFEVQTDKKS      180
           ********************************:*****:********************

c_10      VTAQELDIKARNFLINKKNLYEFNSSPYETGYIKFIENNGNTFWYDMMPAPGDKFDQSKY      240
c_5       VTAQELDIKARNFLINKKNLYEFNSSPYETGYIKFIENNGNTFWYDMMPAPGDKFDQSKY      240
c_11      VTAQELDIKARNFLINKKNLYEFNSSPYETGYIKFIENNGNTFWYDMMPAPGDKFDQSKY      240
c_3       VTAQELDIKARNFLINKKNLYEFNSSPYETGYIKFIENNGNTFWYDMMPAPGDKFDQSKY      240
c_4       VTAQELDIKARNFLINKKNLYEFNSSPYETGYIKFIENNGNTFWYDMMPAPGDKFDQSKY      240
c_2       VTAQELDIKARSFLINKKNLYEFNSSPYETGYIKFIENNGNTFWYDMMPAPGDKFDQSKY      240
c_1       VTAQELDIKARNFLINKKNLYEFNSSPYETGYIKFIENNGNTFWYDMMPAPGDKFDQSKY      240
c_13      VTAQELDIKARNFLINKKNLYEFNSSPYETGYIKFIENNGNTFWYDMMPAPGDKFEQSKY      240
c_7       VTAQELDIKARNFLINKKNLYEFNSSPYETGYIKFIENNGNTFWYDMMPAPGDKFDQSKY      240
c_8       VTAQELDIKARNFLINKKNLYEFNSSPYETGYIKFIENNGNTFWYDMMPAPGDKFDQSKY      240
          ***********.*******************************************:****

c_10      LMMYNDNKTVDSTQFHLDVEISKR--      264
c_5       LMMYNDNKTVDSKSVKIEVHLTTKNG      266
c_11      LMMYNDNKTVDSKRVKIEVHLTTKNG      266
c_3       LMMYNDNKTVDSKSVKIEVHLTTKNG      266
c_4       LMMYNDNKTVDSKSVKIEVHLTTKNG      266
c_2       LMMYNDNKTVDSKSVKIEVHLTTKNG      266
c_1       LMMYNDNKTVDSKSVKIEVHLTTKNG      266
c_13      LMMYNDNKTVDSKSVKIEVHLTTKNG      266
c_7       LMMYNDNKTVDSKSVKIEVHLTTKNG      266
c_8       LMMYNDNKTVDSKSVKIEVHLTTKNG      266
          ************. .:::*.::.:
```

SED

```
d_5      MKKFNILIALLFFTSLVISPLNVKANENIDSVKEKELHKKSELSSTALNNMKHSYADKNP     60
d_3      MKKFNILIALLFFTSLVISPLNVKANENIDSVKEKELHKKSELSSTALNNMKHSYADKNP     60
d_1      MKKFNILIALLFFTSLVISPLNVKANENIDSVKEKELHKKSELSSTALNNMKHSYADKNP     60
d_2      MKKFNILIALLFFTSLVISPLNVKANENIDSVKEKELHKKSELSSTALNNMKHSYADKNP     60
         ************************************************************

d_5      IIGENKSTGDQFLENTLLYKNFFTDLINFEDLLINFNSKEMAQHFKSKNVDVYAIRYSIN     120
d_3      IIGENKSTGDQFLENTLLYKKFFTDLINFEDLLINFNSKEMAQHFKSKNVDVYAIRYSIN     120
d_1      IIGENKSTGDQFLENTLLYKKFFTDLINFEDLLINFNSKEMAQHFKSKNVDVYAIRYSIN     120
d_2      IIGENKSTGDQFLENTLLYKKFFTDLINFEDLLINFNSKEMAQHFKSKNVDVYAIRYSIN     120
         ********************:***************************************

d_5      CYGGEIDKTACTYGGVTPHEGNKLKERKKIPINLWINGVQKEVSLDKVQTDKKNVTVQEL     180
d_3      CYGGEIDKTACTYGGVTPHEGNKLKERKKIPINLWINGVQKEVSLDKVQTDKKNVTVQEL     180
d_1      CYGGEIDKTACTYGGVTPHEGNKLKERKKIPINLWINGVQKEVSLDKVQTDKKNVTVQEL     180
d_2      CYGGEIDRTACTYGGVTPHEGNKLKERKKIPINLWINGVQKEVSLDKVQTDKKNVTVQEL     180
         *******:****************************************************

d_5      DAQARRYLQKDLKLYNNDTLGGKIQRGKIEFDSSDGSKVSYDLFDVKGDFPEKQLRIYSD     240
d_3      DAQARRYLQKDLKLYNNDTLGGKIQRGKIEFDSSDESKVSYDLFDVKGDFPEKQLRIYSD     240
d_1      DAQARRYLQKDLKLYNNDTLGGKIQRGKIEFDSSDGSKVSYDLFDVKGDFPEKQLRIYSD     240
d_2      DAQARRYLQKDLKLYNNDTLGGKIQRGKIEFDSSDGSKVSYDLFDVKGDFPEKQLRIYSD     240
         *********************************** ************************

d_5      NKTLSTEHLHIDIYLYEK      258
d_3      NKTLSTEHLHIDIYLYEK      258
d_1      NKTLSTEHLHIDIYLYEK      258
d_2      NKTLSTEHLHIDIYLYEK      258
         ******************
```

SEE

```
e_1      MKKTAFILLLFIALTLTTSPLVNGSEKSEEINEKDLRKKSELQRNALSNLRQIYYYNEKA     60
e_2      MKKTAFILLLFIALTLTTSPLVNGSEKSEEINEKDLRKKSELQGIALSNLRQIYYYNEKA     60
e_3      MKKTAFILLLFIALTLTTSPLVNGSEKSEEINEKDLRKKSELQGTALSNLRQIYYYNEKA     60
         *******************************************  ***************

e_1      ITENKESDDQFLENTLLFKGFFTGHPWYNDLLVDLGSKDATNKYKGKKVDLYGAYYGYQC     120
e_2      ITENKESDDQFLENTLLFKDFFTGHPWYNDLLVDLGSKDATNKYKGKKVDLYGAYYGYQC     120
e_3      ITENKESDDQFLENTLLFKDFFTGHPWYNDLLVDLGSKDATNKYKGKKVDLYGAYYGYQC     120
         *******************.****************************************

e_1      AGGTPNKTACMYGGVTLHDNNRLTEEKKVPINLWIDGKQTTVPIDKVKTSKKEVTVQELD     180
e_2      AGGTPNKTACMYGGVTLHDNNRLTEEKKVPINLWIDGKQTTVPIDKVKTSKKEVTVQELD     180
e_3      AGGTPNKTACMYGGVTLHDNNRLTEEKKVPINLWIDGKQTTVPIDKVKTSKKEVTVQELD     180
         ************************************************************

e_1      LQARHYLHGKFGLYNSDSFGGKVQRGLIVFHSSEGSTVSYDLFDAQGQYPDTLLRIYRDN     240
e_2      LQARHYLHEKFGLYNSDSFGGKVQRGLIVFHPSEGSTVSYDLFDAQGQYPDTLLRIYRDN     240
e_3      LQARHYLHEKFGLYNSDSFGGKVQRGLIVFHSSEGSTVSYDLFDAQGQYPDTLLRIYRDN     240
         ******** ********************** ****************************

e_1      KTINSENLHIDLYLYTT       257
e_2      KTINSENLHIDLYLYTT       257
e_3      KTINSENLHIDLYLYTT       257
         *****************
```

SEG

```
g_5         MRL---------------F---YIA-------------AIIITLLCLIXGNVMNLYTSPPVE      31
g_14        MKKLSTVIIILILEIVLYNIKYANAQPDPKLDELNKVSDYKINKGTMGNVMNLYMSPPVE      60
g_3         MKKLSTVIIILILEIVFHNMNYVNAQPDPKLDELNKVSDYKNNKGTMGNVMNLYTSPPVE      60
g_7         MKKLSTVIIILILEIVFHNMNYVNAQPDPKLDELNKVSDYKNNKGTMGNVMNLYTSPPVE      60
g_1         MKKLSTVIIILILEIVFHNINYANALPDPKIDELNKVSDYKSNKGTMGNVMNLYMSPPVE      60
g_2         MKKLSTVIIILILEIVFHNINYANAQPDPKIDELNKVSDYKSNKGTMGNVMNLYMSPPVE      60
g_4         MKKLSTVIIILILEIVFHNINYANSQPDPKIDELNKVSDYKSNKGTMGNVMNLYMSPPVE      60
            *:               .   *              .   .      ******* *****

g_5         GRGVINSRQFLSHDLIFPIEYKSYNEVKTELENTELANNYKDKKVDIFGVPYFYTCIIPK      91
g_14        GRGVVNSRQFLSHDLIFPIEYKSYNEVKTELKNTELANNYKGKKVDIFGVPYFYTCIIPK      120
g_3         GRGVINSRQFLSHDLIFPIEYKSYNEVKTELENTELANNYKDKKVDIFGVPYFYTCIIPK      120
g_7         GRGVINSRQFLSHDLIFPIEYKSYNEVKTELENTELANNYKDKKVDIFGVPYFYTCIIPK      120
g_1         GRGVINSRQFLSHDLIFPIEYKSYNEVKTELENIELANNYKGKKVDIFGVPYFYTCIIPK      120
g_2         GRGVINSRQFLSHDLIFPIEYKSYNEVKTELENTELANNYKGKKVDIFGVPYFYTCIIPK      120
g_4         GRGVINSRQFLSHDLIFPIEYKSYNEVKTELENTELANNYKGKKVDIFGVPYFYTCIIPK      120
            ****:**************************:* *******.******************

g_5         SEPDINQNFGGCCMYGGLTFN-----------VQVTIDNRQSLGFTITTNKNMVTIQELD      140
g_14        HEPDINQNFGGCCMYGGLTLNSSENERDKLITVQVTIDNRQSLGFTITTNKNMVTIQELD      180
g_3         SEPDINQNFGGCCMYGGLTFNSSENERDKLITVQVTIDNRQSLGFTITTNKNMVTIQELD      180
g_7         SEPDINQNFGDCCMYGGLTFNSSENERDKLITVQVTIDNRQSLGFTITTNKNMVTIQELD      180
g_1         SEPDINQNFGGCCMYGGLTFNSSENERDKLITVQVTIDNRQSLGFTITTNKNMVTIQELD      180
g_2         SEPDINQNFGGCCMYGGLTFNSSENERDKLITVQVTIDNRQSLGFTITTNKNMVTIQELD      180
g_4         SEPDINQNFGGCCMYGGLTFNSSENERDKLITVQVTIDNRQSLGFTITTNKNMVTIQELD      180
             *********.********:*           ****************************

g_5         YKARHWLTKEKKLYEFDGSAFESGYIKFTEKNNTSFWFDLFPKKELVPFVPYKFLNIYGD      200
g_14        YKARHWLTKEKKLYEFDGSAFESGYIKFTEKNKASIWFDLFPKKELVPFVPYKFLNIYGD      240
g_3         YKARHWLTKEKKLYEFDGSAFESGYIKFTEKNNTSFWFDLFPKKELVPFVPYKFLNIYGD      240
g_7         YKARHWLTKEKKLYEFDGSAFESGYIKFTEKNNTSFWFDLFPKKELVPFVPYKFLNIYGD      240
g_1         YKARHWLTKEKKLYEFDGSAFESGYIKFTEKNNTSFWFDLFPKKELVPFVPYKFLNIYGD      240
g_2         YKARHWLTKEKKLYEFDGSAFESGYIKFTEKNNTSFWFDLFPKKELVPFVPYKFLNIYGD      240
g_4         YKARHWLTKEKKLYEFDGSAFESGYIKFTEKNNTSFWFDLFPKKELVPFVPYKFLNIYGD      240
            ********************************::*:************************

g_5         NKVVDSKSIKXL---NTH      215
g_14        NKVVDSKSIKMEVFLNTH      258
g_3         NKVVDSKSIKMEVFLNTH      258
g_7         NKVVDSKSIKMEVFLNTH      258
g_1         NKVVDSKSIKMEVFLNTH      258
g_2         NKVVDSKSIKMEVFLNTH      258
g_4         NKVVDSKSIKMEVFLNTH      258
            **********     ***
```

SEH

```
h_1        MINKIKILFSFLALLLSFTSYAKAEDLHDKSELTDLALANAYGQYNHPFIKENIKSDEIS      60
h_4        MINKIKILFSFLALLLSFTSYAKAEDLHDKSELTDLALANAYGQYNHPFIKENIKSDEIS      60
h_2        MINKIKILFSFLALLLSFTSYAKAEDLHDKSELTDLALANAYGQYNHPFIKENIKSDEIS      60
h_3        MINKIKILFSFLALLLSFTSYAKAEDLHDKSELTDLALANAYGQYNHPFIKENIKSDEIS      60
           ************************************************************

h_1        GEKDLIFRNQGDSGNDLRVKFATADLAQKFKNKSVDIYGASFYYKCEKISENISECLYGG      120
h_4        GEKDLIFRNQGDSGNDLRVKFATADLAQKFKNKNVDIYGASFYYKCEKISENISECLYGG      120
h_2        GEKDLIFRNQGDSGNDLRVKFATADLAQKFKNKNVDIYGASFYYKCEKISENISECLYGG      120
h_3        GEKDLIFRNQGDSGNDLRVKFATADLAQKFKNKNVDIYGASFYYKCEKISENISECLYGG      120
           *********************************.**************************

h_1        TTLNSEKLAQERVIGANVWVDGIQKETELIRTNKKNVTLQELDIKIRKILSDKYKIYYKD      180
h_4        TILNSEKLAQERVIGANVWVDGIQKETELIRTNKKNVTLQELDIKIRKILSDKYKIYYKD      180
h_2        TTLNSEKLAQERVIGANVWVDGIQKETELIRTNKKNVTLQELDIKIRKILSDKYKIYYKD      180
h_3        TTLNSEKLAQERAIGANVWVDGIQKETELIRTNKKNVTLQELDIKIRKILSDKYKIYYKD      180
           * **********.***********************************************

h_1        SEISKGLIEFDMKTPRDYSFDIYDLKGENDYEIDKIYEDNKTLKSDDISHIDVNLYTKKS      240
h_4        SEISKGLIEFDMKTPRDYSFDIYDLKGENDYEIDKIYEDNKTLKSDDISHIDVNLYTKKK      240
h_2        SEISKGLIEFDMKTPRDYSFDIYDLKGENDYEIDKIYEDNKTLKSDDISHIDVNLYTKKK      240
h_3        SEISKGLIEFDMKTPRDYSFDIYDLKGENDYEIDKIYEDNKTLKSDDISHIDVNLYTKKK      240
           ***********************************************************.

h_1        IINENF  246
h_4        V-----  241
h_2        V-----  241
h_3        V-----  241
           :
```

SEI

```
i_2         MKKFKYSFIVVFILFFNVNDFSYAQGDIGVGNLRNFYTKHDYIDLKGLIDKNLPSANQLE     60
i_12        MKKFKYSFIVVFILFFNVNDFSYAQGDIGVGNLRNFYTKHDYIDLKGLIDKNLPSANQLE     60
i_6         MKKFKYSFILVFILLFNIKDLTYAQGDIGVGNLRNFYTKHDYIDLKGVTDKNLPIANQLE     60
i_15        MKKFKYSFILVFILLFNIKDLTYAQGDIGVGNLRNFYTKHDYIDLKGVTDKNLPIANQLE     60
i_3         MKKFKYSFILVFILLFNIKDLTYAQGDIGVGNLRNFYTKHDYIDLKGVTDKNLPIANQLE     60
i_4         MKKFKYSFILVFILLFNIKDLTYAQGDIGVGNLRNFYTKHDYIDLKGVTDKNLPIANQLE     60
i_8         MKKFKYSFIVVFILFFNVYDLSYAQGDIGVGNLRNFYTKYDYIDLKGVTDKNLPTANQLE     60
i_11        MKKFKYSFIVVFILFFNVYDLSYAQGDIGVGNLRNFYTKYDYIDLKGVTDKNLPTANQLE     60
i_13        MKKFKYSFIVVFILFFNVNDLSYAQGDIGVGNLRNFYTKYDYIDLKGVTDKNLPIANQLE     60
i_17        MKKFKYSFIVVFILFFNVDDLSYAQGDIGVGNLRNFYTKYDYIDLKGVTDKNLPIANQLE     60
i_1         MKKIKYSFILVFILFFNIKDLSYAQGDIGVGNLRNFYTKYDYIDLKGVTDKNLPIANQLE     60
i_5         MKKIKYSFILVFILFFNIKDLSYAQGDIGVGNLRNFYTKYDYIDLKGVTDKNLPIANQLE     60
i_10        MKKIKYSFILFFILFFNIKDLSYAQGDIGVGNLRNFYTKYDYIDLKGVTDKNLPIANQLE     60
            ***:*****:.***:**: *::*****************:*******: ***** *****

i_2         FSTGINDLISESNNWDEISKFKGKKLDIFGIDYNGPCKSKYMYGGATLSGQYLNSARKIP     120
i_12        FSTGINDLISESNNWDEISKFKGKKLDIFGIDYNGPCKSKYMYGGATLSGQYLNSARKIP     120
i_6         FSTGTNDLISESNNWDEISKFKGKKLDIFGIDYNGPCKSKYMYGGATLSGQYLNSARKIP     120
i_15        FSTGTNDLISESNNWDEISKFKGKKLDIFGIDYNGPCKSKYMYGGATLSGQYLNSARKIP     120
i_3         FSTGTNDLISESNNWDEISKFKGKKLDIFGIDYNGPCKSKYMFGGATLSGQYLNSARKIP     120
i_4         FSTGTNDLISESNNWDEISKFKGKKLDIFGIDYNGPCKSKYMYGGATLSGQYLNSARKIP     120
i_8         FSTGPNDLISESNNWDEISKFKGKKLDIFGIDYNGPCKSKYMYGGATLSGQYLNSARKIP     120
i_11        FSTGPNDLISESNNWDEISKFKGKKLDIFGIDYNGPCKSKYMYGGAALSGQYLNSARKIP     120
i_13        FSTATNDLISESNNWDEISKFKGKKLDIFGIDYNGPCKSKYMYGGATLSGQYLNSARKIP     120
i_17        FSTGTNDLISESNNWDEISKFKGKKLDIFGIDYNGPCKSKYMYGGATLSGQYLNSARKIP     120
i_1         FSTGTNDLISESNNWDEISKFKGKKLDIFGIDYNGPCKSKYMYGGVTLSGQYLNSARKIP     120
i_5         FSTGTNDLISESNNWDEISKFKGKKMDIFGIDYNGPCKTKYMYGGATLSGQYLNSARKIP     120
i_10        FSTGTNDLISESNNWDEISKFKGKKMDIFGIDYNGPCKTKYMYGGATLSGQYLNSARKIP     120
```

```
          ***. ********************:************:***:**.:*************

i_2       INLWVNGKHKTISTDKISTNKKLVTAQEIDVKLRRYLQEEYNIYGHNSTGKGKEYGYKSK      180
i_12      INLLVNGKHKTISTDKISTNKKLVTAQEIDVKLRRYLQEEYNIYGHNSTGKGKEYGYKSK      180
i_6       INLWVNGKHKTISTDKIATNKKLVTAQEIDVKLRRYLQEEYNIYG---------------      165
i_15      INLWVNGKHKTISTDKIATNKKLVTAQEIDVKLRRYLQEEYNIYGHNNTGKGKEYGYKSK      180
i_3       INLWVNGKHKTISTDKIATNKKLVTAQEIDVKLRRYLQEEYNIYGHNNTGKGKEYGYKSK      180
i_4       INLWVNGKHKTISTDKIATNKKLVTAQEIDVKLRRYLQEEYNIYGHNNTGKGKEYGYKSK      180
i_8       INLWVNGKHKTISTDKIATNKKLVTAQEIDVKLRRYLQEEYNIYGHNNTGKGKEYGYKSK      180
i_11      INLWVNGKHKTISTDKIATNKKLVTAQEIDVKLRRYLQEEYNIYGHNNTGKGKEYGYKSK      180
i_13      INLWVNGKHKTISTDKIATNKKLVTAQEIDVKLRRYLQEEYNIYGHNNTGKGKEYGYKSK      180
i_17      INLWVNGKHKTISTDKIATNKKLVTAQEIDVKLRRYLQEEYNIYGHNNTGKGKEYGYKSK      180
i_1       INLWVNGKHKTISTDKIATNKKLVTAQEIDVKLRRYLQEEYNIYGHNNTGKGKEYGYKSK      180
i_5       INLWINGKHKTISTDKIATNKKLVTAQEIDVKLRRYLQEEYNIYGHNNNGKGKEYGYKSK      180
i_10      INLWINGKHKTISTDKIATNKKLVTAQEIDVKLRRYLQEEYNIYGHNNNGKGKEYGYKSK      180
          *** :************:***************************

i_2       FYSGFNKGKVLFHLNDEKSFSYDLFYTGDGVPVSFLKIYEDNKIIESEKFHLDVEISYVD      240
i_12      FYSGFNKGKVLFHLNDEKSFSYDLFYTGDGVPVSFLKIYEDNKIIESEKFHLDVEISYVD      240
i_6       ---------------------------GDGLPVSFLKIYEDNKIIESEKFHLDVEISYVD      198
i_15      FYSGFNNGKVLFHLNNEKSFSYDLFYTGDGLPVSFMKIYEDNKIIESEKFHLDVEISYVD      240
i_3       FYSGFNNGKVLFHLNNEKSFSYDLFYTGDGLPVSFLKIYEDNKIIESEKFHLDVEISYVD      240
i_4       FYSGFNNGKVLFHLNNEKSFSYDLFYTGDGLPVSFLKIYEDNKIIESEKFHLDVEISYVD      240
i_8       FYSGFNKGKVLFHLNDEKSFSYDLFYTGDGVPVSFLKIYEDNKIIESEKFHLDVEISYVD      240
i_11      FYSGFNKGKVLFHLNDEKSFSYDLFYTGDGVPVSFLKIYEDNKIIESEKFHLDVEISYVD      240
i_13      FYSGFNKGKVLFHLNDEKSFSYDLFYTGDGVPVSFLKIYEDNKIIESEKFHLDVEISYVD      240
i_17      FYSGFNKGKVLFHLNDEKSFSYDLFYTGDGVPVSFLKIYEDNKIIESEKFHLDVEISYVD      240
i_1       FYSGFNKGKVLFHLNDEKSFSYDLFYTGDGLPVSFLKIYEDNKIIESEKFHLDVEISYVD      240
i_5       FYSGFNKGKVLFHLNDEKSFSYDLFYTGDGLPVSFLKIYEDNKIIESEKFHLDVEISYVD      240
i_10      FYSGFNKGKVLFHLNDEKSFSYDLFYTGDGLPVSFLKIYEDNKIIESEKFHLDVEISYVD      240
                                     ***:****:************************

i_2       SNK    243
i_12      SNK    243
i_6       SN-    200
i_15      SN-    242
i_3       SN-    242
i_4       SN-    242
i_8       SN-    242
i_11      SN-    242
i_13      SN-    242
i_17      SN-    242
i_1       SN-    242
i_5       SN-    242
i_10      SN-    242
          **
```

SEJ

```
sej_4      MKKTIFILIFSLTLTLLITPLVYSDSKNETIKEKNLHKKSELSSITLNNLRHIYFFNEKG     60
sej_2      MKKRIFILIFSLTLTLLITPLVYSDSKNETIKEKNLHKKSELSSITLNNLRHIYFFNEKG     60
sej_1      MKKTIFILIFSLTLTLLITPLVYSDSKNETIKEKNLHKKSELSSITLNNLRHIYFFNEKG     60
sej_3      MKKTIFILIFSLTLTLLITPLVYSDSKNETIKEKNLHKKSELSSITLNNLRHIYFFNEKG     60
           *** ********************************************************

sej_4      ISEKIMTEDQFLDYTLLFKSFFISHSQYNDLLVQFDSKETVNKFKGKQVDLYGSYYGFQC     120
sej_2      ISEKIMTEDQFLDYTLLFKSFFISHSQYNDLLVQFDSKETVNKFKGKQVDLYGSYYGFQC     120
sej_1      ISEKIMTEDQFLDYTLLFKSFFISHSQYNDLLVQFDSKETVNKFKGKQVDLYGSYYGFQC     120
sej_3      ISEKIMTEDQFLDYTLLFKSFFISHSQYNDLLVQFDSKETVNKFKGKQVDLYGSYYGFQC     120
           ************************************************************

sej_4      SGGKSNKTACMYGGVTLHENNQLYDTKKIPINLWIDSIRTVVPLDIVKTNKKKVTIQELD     180
sej_2      SGGKPNKTACMYGGVTLHENNQLYDTKKIPINLWIDSIRTVVPLDIVKTNKKKVTIQELD     180
sej_1      SGGKPNKTACMYGGVTLHENNQLYDTKKIPINLWIDSIRTVVPLDIVKTNKKKVTIQELD     180
sej_3      SGGKPNKTACMYGGVTLHENNQLYDTKKIPINLWIDSIRTVVPLDIVKTNKKKVTIQELD     180
           **** *******************************************************

sej_4      LQARYYLHKQYNLYNPSTFDGKIQKGLIVFHTSKEPLVSYDLFNVIGQYPDKLLKIYQDN     240
sej_2      LQARYYLHKQYNLYNPSTFGGKIQKGLIVFHTSKEPLVSYDLFNVIGQYPDKLLKIYQDN     240
sej_1      LQARYYLHKQYNLYNPSTFDGKIQKGLIVFHTSKEPLVSYDLFNVIGQYPDKLLKIYQDN     240
sej_3      LQARYYLHKQYNLYNPSTFGGKIQKGLIVFHTSKEPLVSYDLFNVIGQYPDKLLKIYQDN     240
           ******************.*****************************************

sej_4      KIIESENMHIDIYLYTSLIVLISLPLVL   268
sej_2      KIIESENMHIDIYLYTSLIVLISLPLVL   268
sej_1      KIIESENMHIDIYLYTSLIVLISLPLVL   268
sej_3      KIIESENMHIDIYLYTSLIVLISLPLVL   268
           ****************************
```

SEK

```
k_2      MKKLISILLINIIILGVSNNASAQGDIGIDNLRNFYTKKDFINLKDVKDNDTPIANQLQF     60
k_9      MKKLISILLINIIILGVSNNASAQGDIGIDNLRNFYTKKDFINLKDVKDNDTPIANQLQF     60
k_7      MKKLIVIILINIITLSVSNSASAQGDIGIDNLRNFYTKKDFVDLKDVKDNDTPIANQLQF     60
k_4      MKKLISILLINIIILGVSNSASAQGDIGIDNLRNFYTKKDFIDLKDVKDNDTPIANQLQF     60
k_1      MKKLISILLINIIILGVSNSASAQGDIGIDNLRNFYTKKDFVDLKDVKDNDTPIANQLQF     60
k_3      MKKLISILLINIIILGVSNSASAQGDIGIDNLRNFYTKKDFVDLKDVKDNDTPIANQLQF     60
k_5      MKKLISILLINIIILGVSNNASAQGDIGIDNLRNFYTKKDFVDLKDVKDNDTPIANQLQF     60
k_8      MKKLISILLINIIILGVSNNASAQGDIGIDNLRNFYTKKDFVDLKDVKDNDTPIANQLQF     60
```

```
          ***** *:***** *.***.*********************::*****************

k_2       SNESYDLISESKDFNKFSNFKGKKLDVFGISYNGQCNTKYIYGGITATNEYLDKPRNIPI       120
k_9       SNESYDLISESKDFNKFSNFKGKKLDVFGISYNGQCNTKYIYGGITATNEYLDKPRNIPI       120
k_7       SNESYDLISESKDFNKLSNFKGKKLDVFGISYNGQCNNKYIYGGVTATNEYLDKPRNIPI       120
k_4       SNESYDLISESKDFNKFSNFKGKKLDVFGISYNGQCNTKYIYGGVTATNEYLDKSRNIPI       120
k_1       SNESYDLISESKDFNKFSNFKGKKLDVFGISYNGQCNTKYIYGGVTATNEYLDKSRNIPI       120
k_3       SNESYDLISESKDFNKFSNFKGKKLDVFGISYNGQCNTKYIYGGVTATNEYLDKSRNIPI       120
k_5       SNESYDLISESKDFNKFSNFKGKKLDVFGISYNGQCNTKYIYGGVTATNEYLDKSRNIPI       120
k_8       SNESYDLISESKDFNKFSNFKGKKLDVFGISYNGQCNTKYIYGGVTATNEYLDKSRNIPI       120
          ****************:********************.******:********* *****

k_2       NIWINGNHKTISTNKVSTNKKFVTAQEIDIKLRRYLQEEYNIYGHNGTKKGEEYGHKSKF       180
k_9       NIWINGNHKTISTNKVSTNKKFVTAQEIDIKLRRYLQEEYNIYGHNGTKKGEEYGHKSKF       180
k_7       NIWINGNHKTISTNKVSTNKKFVTAQEIDVKLRKYLQEEYNIYGHNGTKKGEEYGHKSKF       180
k_4       NIWINGNHKTISTNKVSTNKKFVTAQEIDVKLRKYLQEEYNIYGHNGTKKGEEYGHKSKF       180
k_1       NIWINGNHKTISTNKVSTNKKFVTAQEIDVKLRKYLQEEYNIYGHNGTKKGEEYGHKSKF       180
k_3       NIWINGNHKTISTNKVSTNKKFVAAQEIDVKLRKYLQEEYNIYGHNGTKKGEEYGHKSKF       180
k_5       NIWINGNHKTISTNKVSTNKKLVTAQEIDVKLRKYLQEEYNIYGHNGTKKGEEYGHKSKF       180
k_8       NIWINGNHKTISTNKVSTNKKLVTAQEIDVKLRKYLQEEYNIYGHNGTKKGEEYGHKSKF       180
          *********************:*:*****:***:**************************

k_2       YSGFNIGKVTFHLNNNDTFSYDLFYTGDDGLPKSFLKIYEDNKTVESEKFHLDVDISYKE       240
k_9       YSGFNIGKVTFHSNNNDTFSYDLFYTGDDGLPKSFLKIYEDNKTVESEKFHLDVDISYKE       240
k_7       YSGFNIGKVTFHLNNNDTFSYDLFYTGDDGLPKSFLKIYEDNKTVESEKFHLDVDISYKE       240
k_4       YSGFNIGKVTFHLNNNDTFSYDLFYTGDDGLPKSFLKIYEDNKTVESEKFHLDVDISYKE       240
k_1       YSGFNIGKVTFHLNNNDTFSYDLFYTGDDGLPKSFLKIYEDNKTVESEKFHLDVDISYKE       240
k_3       YSGFNIGKVTFHLNNNDTFSYDLFYTGDDGLPKSFLKIYEDNKTVESEKFHLDVDISYKE       240
k_5       YSGFNIGKVTFHLNNNDTFSYDLFYTGDDGLPKSFLKIYEDNKTVESEKFHLDVDISYKE       240
k_8       YSGFNIGKVTFHLNNNDTFSYGLFYTGDDGLPKSFLKIYEDNKTVESEKFHLDVDISYKE       240
          ************ ********.**************************************

k_2       TK      242
k_9       TK      242
k_7       TI      242
k_4       TI      242
k_1       TI      242
k_3       TI      242
k_5       TI      242
k_8       TI      242
          *
```

SEL

```
l_13       MKKRLLFVIVITLFIFSSNHTVLSNGDVGPGNLRNFYTKYEYVNLKNIKDKNSPESHRLE       60
l_12       MKKRLLFVIVITLFIFSSNHTVLSNGDVGPGNLRNFYTKYEYVNLKNVKDKNSPESHRLE       60
l_9        MKKRLLFVIVITLFIFSSNHTVLSNGDVGPGNLRNFYTKYEYVNLKNVKDKNSPESHRLE       60
l_8        MKKRLLFVIVITLFIFSSNHTVLSNGDVGPGNLRNFYTKYEYVNLKNVKDKNSPESHRLE       60
l_7        MKKRLLFVIVITLFIFSSNHTVLSNGDVGPGNLRNFYTKYEYVNLKNVKDKNSPESHRLE       60
l_6        MKKRLLFVIVITLFIFSSNHTVLSNGDVGPGNLRNFYTKYEYVNLKNVKDKNSPESHRLE       60
l_4        MKKRLLFVIVITLFIFSSNHTVLSNGDVGPGNLRNFYTKYEYVNLKNVKDKNSPESHRLE       60
l_1        MKKRLLFVIVITLFIFSSNHTVLSNGDVGPGNLRNFYTKYEYVNLKNVKDKNSPESHRLE       60
l_2        MKKRLLFVIVITLFIFSSNHTVLSNGDVGPGNLRNFYTKYEYVNLKNVKDKNSPESHRLE       60
```

```
l_5         MKKRLLFVIVITLFIFSSNHTVLSNGDVGTGNLRNFYTKYEYVNLKNVKDKNSPESHRLE    60
l_10        ------------------------------------------MNLKNVKDKNSPESHRLE    18
                                                      :****:************

l_13        YSYKNDTLYAEFDNEYITSDLKGKNVDVFGISYKYGYNSRTIYGGVTKAENNKLDSPRII    120
l_12        YSYKNDTLYAEFDNEYITSDLKGKNVDIFGISYKYGSNSRTIYGGVTKAENNKLGSPRII    120
l_9         YSYKNDTLYAEFDNEYITSDLKGKNVDVFGISYKYGSNSRTIYGGVTKAENNKLDSPRII    120
l_8         YSYKNDTLYAEFDNEYITSDLKGKNVDIFGISYKYGSNSRTIYGGVTKAENNKLDSPRII    120
l_7         YSYKNDTLYAEFDNEYITSDLKGKNVDVFGISYKYGSNSRTIYGGVTKAENNKLDSPRII    120
l_6         YSYKNDTLYAEFENEYITSDLKGKNVDVFGISYKYGSNSRTIYGGVTKAENNKLDSPRII    120
l_4         YSYKNDTLYAEFDNEYITSDLKGKNVDVFGISYKYGSNSRTIYGGVTKAENNKLDSPRII    120
l_1         YSYKNDTLYAEFDNEYITSDLKGKNVDVFGISYKYGSNSRTIYGGVTKAENNKLDSPRII    120
l_2         YSYKNDTLYAEFDNEYITSDLKGKNVDVFGISYKYGYNSRTIYGGVTKAENNKLDSPRII    120
l_5         YSYKNDTLYAEFDNEYITSDLKGKNVDVFGISYKYGSNSRTIYGGVTKAENNKLDSPRII    120
l_10        YSYKNDTLYAEFDNEYITSDLKGKNVDVFGISYKYGSNSRTIYGGVTKAENNKLDSPRII    78
            ************:**************:******** *****************.*****

l_13        PINLIINGKHQTVTTKSVSTDKKMVTAQEIDVKLRKYLQDEFNIYGHNDTGKGKEYGTSS    180
l_12        PINLIINGKHQTVTTKSVSTDKKMVTAQEIDVKLRKYLQDEFNIYGHNDTGKGKEYGTSS    180
l_9         PINLIINGKHQTVTTKSVSTDKKMVTAQEIDVKLRKYLQDEFNIYGHNDTGKGKEYGTSS    180
l_8         PINLIINGKHQTVTTKSVSTDKKMVTAQEIDVKLRKYLQDEFNIYGHNDTGKGKEYGTSS    180
l_7         PINLIINGKHQTVTTKSVSTDKKMVTAQEIDVKLRKYLQDEFNIYGHNDTGKGKEYGTSS    180
l_6         PINLIINGKHQTVTTKSVSTDKKMVTAQEIDVKLRKYLQDEFNIYGHNDTGKGKEYGTSS    180
l_4         PINLIINGKHQTVTTKCVSTDKKMVTAQEIDVKLRKYLQDEFNIYGHNDTGKGKEYGTSS    180
l_1         PINLIINGKHQTVTTKSVSTDKKMVTAQEIDVKLRKYLQDEFNIYGHNDTGKGKEYGTSS    180
l_2         PINLIINGKHQTVTTKSVSTDKKMVTAQEIDVKLRKYLQDEFNIYGHNDTGKGKEYGTSS    180
l_5         PINLIINGKHQTVTTKSVSTDKKMVTAQEIDVKLRKYLQDEFNIYGHNDTGKGKEYGTSS    180
l_10        PINLIINGKHQTVTTKSVSTDKKMVTAQEIDVKLRKYLQDEFNIYGHNDTGKGKEYGTSS    138
            ****************.*******************************************

l_13        KFYSGFDKGSVVFHMNDGSNFSYDLFYTGYGLPESFLKIYKDNKTVDSTQFHLDVEISKR    240
l_12        KFYSGFDKGSVVFHMNDGSNFSYDLFYTGYGLPESFLKIYKDNKTVDSTQFHLDVEISKR    240
l_9         KFYSGFDKGSVVFHINDGSNFSYDLFYTGYGLPESFLKIYKDNKTVDSTQFHLDVEISKR    240
l_8         KFYSGFDKGSVVFHMNDGSNFSYDLFYTGYGLPESFLKIYKDNKTVDSTQFHLDVEISKR    240
l_7         KFYSGFDKGSVVLHMNDGSNFSYDLFYTGYGLPESFLKIYKDNKTVDSTQFHLDVEISKR    240
l_6         KFYSGFDKGSVVFHMNDGSNFSYDLFYTGYGLPESFLKIYKDNKTVDSTQFHLDVEISKR    240
l_4         KFYSGFDKGSVVFHMNDGSNFSYDLFYTGYGLPESFLKIYKDNKTVDSTQFHLDVEISKR    240
l_1         KFYSGFDKGSVVFHMNDGSNFSYDLFYTGYGLPESFLKIYKDNKTVDSTQFHLDVEISKR    240
l_2         KFYSGFDKGSVVFHMNDGSNFSYDLFYTGYGLPESFLKIYKDNKTVDSTQFHLDVEISKR    240
l_5         KFYSGFDKGSVVFHMNDGSNFSYDLFYTGYGLPESFLKIYKDNKTVGSTQFHLDVEISKR    240
l_10        KFYSGFDKGSVVFHMNDGSNFSYDLFYTGYGLPESFLKIYKDNKTVGSTQFHLDVEISKR    198
            ************:*:*******************************.*************
```

SEM

```
m_3         MKRILIIVVLLFCYSQNHIATADVGVLNLRNYYGSYPIEDHQSINPENNHLSHQLVFSMD    60
m_8         MKRILIIVVLLFCYSQNHIATADVGVLNLRNYYGSYPIEDHQSINPENNHLSHQLVFSMD    60
m_15        MKRILIIVVLLFCYSQNHIATADVGVLNLRNYYGSYPIEDHQSINPENNHLSHQLVFSMD    60
m_4         MKRILIIVVLLFCYSQNHIATADVGVLNLRNYYGSYPIEDHQSINPENNHLSHQLVFSMD    60
m_6         MKRILIIVVLLFCYSQNHIATADVGVLNLRNYYGSYPIEDHQSINPENNHLSHQLVFSMD    60
m_1         MKRILIIVVLLFCYSQNHIATADVGVLNLRNYYGSYPIESHQNINPDNNSLSHQLVFSKD    60
m_5         MKRILIIVVLLFCYSQNHIATADVGVLNLRNYYGSYPIEDHQNINPDNNRLSHQLVFSKD    60
m_2         MKRILIIVVLLFCYSQNHIATADVGVLNLRNYYGSYPIEDHQNINPDNNRLSHQLVFSMD    60
m_9         MKRILIIVVLLFCYSQNHIATADVGVLNLRNYYGSYPIEDHQNINPDNNRLSHQLVFSMD    60
m_19        MKRILIIVVLLFCYSQNHIATADVGVLNLRNYYGSYPIEDHQNINPDNNRLSHQLVFSMD    60
```

```
          **************************************.**.***:** ******** *

m_3       NSSITAEFKNVDDVKKFKNHAVDVYGLSYSGYCLKNKYIYGGVTLAGDYLEKSRCIPINL      120
m_8       NSTITAEFKNVDDVKKFKNHAVDVYGLSYSGYCLKNKYIYGGVTLAGDYLEKSRCIPINL      120
m_15      NSTVTAEFKNVDDVKKFKNHAVDVYGLSYSGYCLKNKYIYGGVTLAGDYLEKSRRIPINL      120
m_4       NSTVTAEFKNVDDVKKFKNHAVDVYGLSYSGYCLKNKYIYGGVTLAGDYLEKSRRIPINL      120
m_6       NSTVTAEFKNVDDVKKFKNHAVDVYGLSYSGYCLKNKYIYGGVTLAGDYLEKSRRIPINL      120
m_1       NSTVTAEFKNVEDVKKFKNRAVDVYGLSYSGYCLKNKYMYGGVTLAGDYLEKSRCIPINL      120
m_5       NSTVTAEFKNVEDVKKFKNRAVDVYGLSYSGYCLKNKYMYGGVTLAGDYLEKSRCIPINL      120
m_2       NSTVTAEFKNVEDVKKFKNRAVDVYGLSYSGYCLKNKYMYGGVTLAGDYLEKSRCIPINL      120
m_9       NSTVTAEFKNVDDVKKFKNRAVDVYGLSYSGYCLKNKYMYGGVTLAGDYLEKSKYIPINL      120
m_19      NSTVTAEFKNVDDVKKFKNRAVDVYGLSYSGYCLKNKYMYGGVTLAGDYLEKSKYIPINL      120
          **::*******:*******:*****************:**************: *****

m_3       WVNGEHQTISTDKVSTNKKLVTAQEIDTKLRRYLQEEYNIYGFNDTNKGRNYGNKSKFSS      180
m_8       WVNGEHQTISTDKVSTNKKLVTAQEIDTKLRRYLQEEYNIYGFNDTNKGRNYGNKSKFSS      180
m_15      WVNGEHQTISTDKVSTNKKLVTAQEIDTKLRRYLQEEYNIYGFNDTNKVRNYGNKSKFSS      180
m_4       WVNGEHQTISTDKVSTNKKLVTAQEIDTKLRRYLQEEYNIYGFNDTNKGRNYGNKSKFSS      180
m_6       WVNGEHQTISTDKVSTNKKLVTAQEIDTKLRRYLQEEYNIYGFNDTNKGRNYGNKSKFSS      180
m_1       WVNGNHKTISTDKVSTNKKIVTAQEIDTKLRRYLQEEYNIYGFNDTNKGRNYGTKSKFFS      180
m_5       WVNGNLKTISTDKVSTNKKIVTAQEIDTKLRRYLQEEYNIYGFNDTNKGRNYGTKSKFFS      180
m_2       WVNGEHQTISTEKVSTNKKIVTAQEIDTKLRRYLQEEYNIYGFNDTNKGRNYGTKSKFSS      180
m_9       WVNSEHQTISTEKVSTNKKIVTAQEIDTKLRRYLQEEYNIYGFNDTNKGRNYGTKSKFSS      180
m_19      WVNSEHQTISTEKVSTNKKIVTAQEIDTKLRRYLQEEYNIYGFNDTNKGRNYGTKSKFSS      180
          ***.: :****:*******:**************************** ****.**** *

m_3       GFNAGKILFHLNDGSSFSYDLFDTGTGQAESFLKIYNDNKTVETEKFHLDVEISYKDES       239
m_8       GFNAGKILFHLNDGSSFSYDLFDTGTGQAESFLKIYNDNKTVETEKFHLDVEISYKDES       239
m_15      GFNAGKILFHLNDGSSFSYDLFDTGTGQAESFLKIYNDNKTVETEKFHLDVEISYKDES       239
m_4       GFNAGKILFHLNDGSSFSYDLFDTGTGQAESFLKIYNDNKTVETEKFHLDVEISYKDES       239
m_6       GFNAGKILFHLNDSSSFSYDLFDTGTGQAESFLKIYNDNKTVETEKFHLDVEISYKDES       239
m_1       GFNTGKISFHLNDGTSFSYDLFDTGTGQAESFLKIYNDNKTVETDKFHLDVEISYKDES       239
m_5       GFNTGKISFHLNDGTSFSYDLFDTGTGQAESFLKIYNDNKTVETDKFHLDVEISYKDES       239
m_2       GFNTGKISFHLNDGSSFSYDLFDTGTGQAESFLKIYNDNKTVETDKFHLDVEISYKDES       239
m_9       GFNTGKVSFHLNDGSSFSYDLFDTGTGQAESFLKIYNDNKTVETDKFHLDVEISYKDES       239
m_19      GFNTGKISFHLNDGSSFSYDLFDTGTGQAESFLKIYNDNKTVETDKFHLDVEISYKDES       239
          ***:**: *****.:****************************:**************
```

SEN

```
n_4       MRLFCIAAIIITLLCLINNNYVNADVDKNDLKKKSDIDSSKLFNLTSYYTDITWQLDESN      60
n_5       ------------MKCLINNNYVNAEVDKKDLKKKSDLDSSKLFNLTSYYTDITWQLDESN      48
n_3       MRLFYIAAIIITLLCLINNNYVNAEVDKKDLKKKSDLDSSKLFNLTSYYTDITWQLDESN      60
n_7       MRLFYIAAIIITLLCLINNNYVNAEVDKKDLKKKSDLDSSKLFNLTSYYTDITWQLDESN      60
n_8       MRLFYIAVIIITLLCLINNNYVNAEVDKKVLKKKSDIDSSK-----CYYTDITWQLDESN      55
n_6       MRLFYIAVIIITLLCLINNKYVNAEVDKKVLKKKSDIDSSNLFNLTSYYTDITWQLDESN      60
n_1       MRLFYIAVIIITLLCLINNNYVNAEVDKKVLKKKSDIDSSNLFNLTSYYTDITWQLDESN      60
n_2       MRLFYIAVIIITLLCLINNNYVNAEVDKKVLKKKSDIDSSKLFNLTSYYTDITWQLDESN      60
n_9       ------------MLCFLNNNYVNADVNKNNLKKKSELDSSKLFNLANYYTDVTWQLEKSN      48
n_13      MRLFYIALIIITLLCLINNNYVNADVDKNDLKKKSDLDSSKLFNLTSYYTDITWQLEKSN      60
                      : *::**:****:*:*: *****::***:      ****:****::**

n_4       KISTDQLLNNTIILKDIDISVLKTSSLKVEFNSSDLANQFKGKNIDIYGLYYGNKCVGLT      120
```

```
n_5         KISTDQLLNNTIILKNIDISVLKTSSLKVEFNSSDLANQFKGKNIDIYGLYFGNKCVGLT       108
n_3         KISTDQLLNNTIILKNIDISVLKTSSLKVEFNSSDLANQFKGKNIDIYGLYFGNKCVGLT       120
n_7         KISTDQLLNNTIILKNIDISVLKTSSLKVEFNSSDLANQFKGKNIDIYGLYFGNKCVGLT       120
n_8         KISTDQLLNNTIILKNIDISVLKTSSLKVEFNSADLANQFKGKNIDIYGLYYGNKCVGLT       115
n_6         KISTDQLLNNTIILKNIDISVLKTSSLKVEFNSADLANQFKGKNIDIYGLYYGNKCVGLT       120
n_1         KISTDQLLNNTIILKNIDISVLKTSSLKVEFNSADLANQFKGKNIDIYGLYYGNKCVGLT       120
n_2         KISTDQLLNNTIILKNIDISVLKTSSLKVEFNSADLANQFKGKNIDIYGLYYGNKCVGLT       120
n_9         VISSDQLLNNTIIFKNIVISVLNTSSLKVEFNSLDLANQYKGRNVDIFGLYYGNKCIGLH       108
n_13        VISSDQLLNNTIIFKNIVIAELNTSSLKVEFNSLDLANQYKGSNVDIFGLYYGNKCVGLH       120
             **:*********:*:* *: *:********** *****:** *:**:***:****:**

n_4         EEKTSCLYGGVTIYDGNQLDEERVIGVNVFKDGIQQEGFVIKTKKAKVTVQELDTKVRFK       180
n_5         EEKTSCLYGGVTIHDGNQLDEEKVIGVNVFKDGVQQEGFVIKTKKAKVTVQELDTKVRFK       168
n_3         EEKTSCLYGGVTIHDGNQLDEEKVIGVNVFKDGVQQEGFVIKTKKAKVTVQELDTKVRFK       180
n_7         EEKTSCLYGGVTIHDGNQLDEERVIGVNVFKDGVQQEGFVIKTKKAKVTVQELDTKVRFK       180
n_8         EEKTSCLYGGVTIHDGNQLDEEKVIGVNVFKDGVQQEGFVIKTKKAKVTVQELDTKVRFK       175
n_6         EEKTSCLNGGVTIHDGNQLDEEKVIGVNVFKDGVQQEGFVIKTKKAKVTVQELDTKVRFK       180
n_1         EEKTSCLYGGVTIHDGNQLDEEKVIGVNVFKDGVQQEGFVIKTKKAKVTVQELDTKVRFK       180
n_2         EEKTSCLYGGVTIHDGNQLDEEKVIGVNVFKDGVQQEGFVIKTKKAKVTVQELDTKVRFK       180
n_9         GEKTSCLYGGVTIHDGNQLDEERVIGVNVFKDDAQQEGFVIKTKKAKVTVQELDTKVRFK       168
n_13        GEKTSCLYGGVTIHDGNQLDEERVIGVNVFKDDAQQEGFVIKTKKAKVTVQELDTKVRFK       180
             ****** *****:********:*********. **************************

n_4         LENLYKIYNKDTGNIQKGCIFFHSNNHQNQSFYYDLYNIKGSVGAEFFQFYSDNRTVSSS       240
n_5         LENLYKIYNKDTGNIQKGCIFFHSHNHQDQSFYYDLYNVKGSVGAEFFQFYSDNRTVSSS       228
n_3         LENLYKIYNKDTGNIQKGCIFFHSHNHQDQSFYYDLYNVKGSVGAEFFQFYSDNRTVSSS       240
n_7         LENLYKIYNKDTGNIQKGCIFFHSHNHQDQSFYYDLYNVKGSVGAEFFQFYSDNRTVSSS       240
n_8         LENLYKIYNKDTGNIQKGCIFFHSHNQQDQSFYYDLYNIKGSVGAEFFQFYSDNRTVSSS       235
n_6         LENLYKIYNKDTGNIQKGCIFFHSHNHQDQSFYYDLYNIKGSVGAEFFQFYSDNRTVSSS       240
n_1         LENLYKIYNKDTGNIQKGCIFFHSHNHQDQSFYYDLYNIKGSVGAEFFQFYSDNRTVSSS       240
n_2         LENLYKIYNKDTGNIQKGCIFFHSHNQQDQSFYYDLYNIKGSVGAEFFQFYSDNRTVSSS       240
n_9         LENLYKIYNKDTGNIQKGCIFFHSHNNQDHSFYYDLYNIKGSVGAEFFQFYGDNRTVNSS       228
n_13        LENLYKIYNKDTGNIQKGCIFFHSHNNQDHSFYYDLYNIKGSVGAEFFQFYGDNRTVNSS       240
            ************************:*:*::********:************.*****.**

n_4         NYHIDVFLYKD    251
n_5         NYHIDVFLYKD    239
n_3         NYHIDVFLYKD    251
n_7         NYHIDVFLYKD    251
n_8         NYHIDVFLYKD    246
n_6         NYHIDVFLYKD    251
n_1         NYHIDVFLYKD    251
n_2         NYHIDVFLYKD    251
n_9         NYHIDVFLYKD    239
n_13        NYHIDVFLYKD    251
            ***********
```

SEO

```
o_9         MLNVILLIINLIAICNVNNAYANEENPKIEDLCKKSSVDPIALHNIEKDYVNNRFTIDKS       60
o_20        MLNVLLLIINLIAICSVNNAYANEEKPKIEDLCKKSSVDPIALHNIEKDYVNNRFTIDKS       60
o_4         MLNVLLLIINLIAICSVNNAYANEENPKIEDLCKKSSVDDIALHNIDKDYMTNRFTINES       60
o_5         MLNVLLLIIYLIAICSVNNAYANEENPKIEDLCKKSSVDDIALHNIDKDYMTNRFTINES       60
o_1         MLNVLLLIIYLIAICSVNNAYANEENPKIEDLCKKSSVDDIALHNIDKDYMTNRFIINES       60
o_19        MLNVLLLIIYLIAICSVNNAYANEENPKIEDLCKKSSVDDIALHNIDKDYMTNRFIINES       60
o_8         MLNVLLLILNLIAICSVNNAYANEEDPKIESLCKKSSVDPIALHNINDDYINNRFTTVKS       60
o_2         MLNVLLLIIYLIAICSVNNAYANEENPKIEDLCKKSSVDSTALHNINDDYINNRFTTVKS       60
o_7         MLNVLLLIIYLIAICSVNNAYANEENPKIEDLCKKSSVDSTALHNINDDYINNRFTTVKS       60
o_21        MLNVLLLILNLIAICSVNNAYANEEDPKIESLCKKSSVDPIALHNINDDYINNRFTTVKS       60
o_14        MLNVLLLILNLIAICSVNNAYANEEDPKIESLCKKSSVDPIDLHNINDDYINNRFTTVKS       60
o_3         MLNVLLLILNLIAICSVNNAYANEEDPKIESLCKKSSVDPIALHNINDDYINNRFTTVKS       60
```

```
o_6         MLNVLLLILNLIAICSVNNAYANEEDPKIESLCKKSSVDPIALHNINDDYINNRFTTVKS      60
            ****:***: *****.*********.****.********   ****:.**:.***   :*

o_9         PVSTTEKFLDFDLLFKNFTWLDGKSAEFKDLKVEFSSSEISKEYFGKTVDIYGVYYKAHC      120
o_20        PVSTTEKFLDFDLLFKNFTWLDGKSAEFKDLKVEFSSSEISKEYFGKTVDIYGVYYKAHC      120
o_4         TVLTTEKFLDIDLLFKNFTWLDGKSAEFKDLKVEFSSSEISKEYFGKTVDIYGVYYKAHC      120
o_5         PVLTTEKFLDFDLLFKNFTWLDGKSAEFKDLKVEFSSSEISKEYFGKTVDIYGVYYKAHC      120
o_1         PVLTTKKFLDFDLLFKNFTWLDGKSAEFKDLKVEFSSSEISKEYFGKTVDIYGVYYKAHC      120
o_19        PVLTTEKFLDFDLLFKNFTWLDGKSAEFKDLKVEFSSSEISKEYFGKTVDIYGVYYKAHC      120
o_8         IVSTTEKFLDFDLLFKSINWLDGISAEFKDLKVEFSSSAISKEFLGKTVDIYGVYYKAHC      120
o_2         IVSTTEKFLDFDLLFKSINWLDGKSAEFKDLKVEFSSSEISKEYFGKTVDIYGVYYKAHC      120
o_7         IVSTTEKFLDFDLLFKSINWLDGKSAEFKDLKVEFSSSEISKEYFGKTVDIYGVYYKAHC      120
o_21        IVSTTEKFLDFDLLFKSINWLDGISAKFKDLKVEFSSSAISKEFLGKTVDIYGVYYKAHC      120
o_14        IVSTTEKFLDFDLLFKSINWLDGISAEFKDLKVEFSSSAISKEFLGKTVDIYGVYYKAHC      120
o_3         IVSTTEKFLDFDLLFKSINWLDGISAEFKDLKVEFSSSAISKEFLGKTVDIYGVYYKAHC      120
o_6         IVSTTEKFLDFDLLFKSINWLDGISAKFKDLKVEFSSSAISKEFLGKTVDIYGVYYKAHC      120
             * **:****:*****.:.**** **:*********** ****::***************

o_9         HGEHQVKTACTYGGITSHENNKLSEPKNIGVAVYKDNVNVNTFIVTTDKKKVTAQELDIK      180
o_20        HGEHQVKTACTYGGITSHENNKLSEPKNIGVAVYKDNVNVNTFIVTTDKKKVTAQELDIK      180
o_4         HGEHQVKTACTYGGVTPHENNKLSEPKEIGVAVYKDNVNVNTFIVTTDKKKVTAQELDIK      180
o_5         HGEHQVKTACTYGGVTPHENNKLNEPKEIGVAVYKDNVNVNTFIVTTDKKKVTAQELDIK      180
o_1         HGEHQVKTACTYGGVTPHENNKLNEPKEIGVAVYKDNVNVNTFIVTTDKKKVTAQELDIK      180
o_19        HGEHQVKTACTYGGVTPHENNKLNEPKEIGVAVYKDNVNVNTFIVTTDKKKVTAQELDIK      180
o_8         HGEHQVDTACTYGGVTPHENNKLSEPKNIGVAVYKDNVNVNTFIVTTDKKKVTAQELDIK      180
o_2         HGEHQMNTACTYGGVTPHENNKLSEPKNIGVAVYKDNVNVNTFIVTTDKKKVTAQELDIK      180
o_7         YGEHQMNTACTYGGVTPHENNKLSEPKNIGVAVYKDNVNVNTFIVTTDKKKVTAQELDIK      180
o_21        HGEHQVDTACTYGGVTPHENNKLSEPKNIGVAVYKDNVNVNTFIVTTDKKKVTAQELDIK      180
o_14        HGEHQVDTACTYGGVTPHENNKLSEPKNIGVAVYKDNVNVNTFIVTTDKKKVTAQELDIK      180
o_3         HGEHQVDTACTYGGVTPHENNKLSEPKNIGVAVYKDNVNVNTFIVTTDKKKVTAQELDIK      180
o_6         HGEHQVDTACTYGGVTPHENNKLSEPKNIGVAVYKDNVNVNTFIVTTDKKKVTAQELDIK      180
            :****:.*******:* ******.***:********************************

o_9         VRTKLNNEYKLYDRMTSDVQKGYIKFHSHSEQKESFYYDLFYIKGNLPDQYLQIYNDNKT      240
o_20        VRTKLNNEYKLYDRMTSDVQKGYIKFHSHSEQKESFYYDLFYIKGNLPDQYLQIYNDNKT      240
o_4         VRTKLNNVYKLYDRMTSDVQKGYIKFHSHSEHKESFYYDLFYIKGNLPDQYLQIYNDNKT      240
o_5         VRTKLNNVYKLYDRMTSDVQKGYIKFHSHSEHKESFYYDLFYIKGNLPDQCLQIYNDNKT      240
o_1         VRTKLNNVYKLYDRMTSDVQKGYIKFHSHSEHKESFYYDLFYIKGNLPDQYLQIYNDNKT      240
o_19        VRTKLNNVYKLYDRMTSDVQKGYIKFHSHSEHKESFYYDLFYIKGNLPDQYLQIYNDNKT      240
o_8         VRTKLNNAYKLYDRMTSDVQKGYIKFHSHSEHKESFYYDLYNVKGSVGAEFFQFYSDNRT      240
o_2         VRTKLNNAYKLYDRMTSDVQKGYIKFHSHSEHKESFYYDLFYIKGNLPDQYLQIYNDNKT      240
o_7         VRTKLNNAYKLYDRMTSDVQKGYIKFHSHSEHKESFYYDLFYIKGNLPDQYLQIYNDNKT      240
o_21        VRTKLNNAYKLYDRMTSDVQKGYIKFHSHSEHKESFYYDLFYIKGNLPDQYLQIYNDNKI      240
o_14        VRTKLNNAYKLYDRMTSDVQKGYIKFHSHSEHKESFYYDLFYIKGNLPDQYLQIYNDNKT      240
o_3         VRTKLNNAYKLYDRMTSDVQKGYIKFHSHSEHKESFYYDLFYIKGNLPDQYLQIYNDNKT      240
o_6         VRTKLNNAYKLYDRMTSDVQKGYIKFHSHSEHKESFYYDLFYIKGNLPDQYLQIYNDNKT      240
            ******* ***********************:********: :**.:  : :*:*.**:

o_9         IDSSDYHIDVYLFT- 254
o_20        IDSSDYHIDVYLFT- 254
o_4         IDSSDYHIDVYLFT- 254
o_5         IDSSDYHIDVYLFT- 254
o_1         IDSSDYHIDVYLFT- 254
o_19        IDSSDYHIDVYLFT- 254
o_8         VSSSNYHIDVFLYKD 255
o_2         IDSSDYHIDVYLFT- 254
o_7         IDSSDYHIDVYLFT- 254
o_21        IDSSDYHIDVYLFT- 254
o_14        IDSSDYHIDVYLFT- 254
```

```
o_3      IDSSDYHIDVYLFT- 254
o_6      IDSSDYHIDVYLFT- 254
         :.**:*****:*:.
```

SEP

```
p_4      MSKIKKTTFILLSFIALTLITSPFVNCSEKSEEINGKDLQKKSELQGTALSNLRQTYYHN      60
p_2      MSKIKKTTFILLSFIALTLITSPFVNCSEKSEEINGKDLQKKSELQGTALSNLRQTYYHN      60
p_3      MSKIKKITFILLSFIALTLITSPFVNCSEKSEEINGKDLQKKSELQGTALSNLRQTYYHN      60
         ****** *****************************************************

p_4      GSAIIENKESNDQFLKNTILFNDFFTGHQWYNDLLVDLGSKDTANIYKGKKVDLYGVYYG      120
p_2      GSAIIENKESNDQFLKNTILFNDFFTGHQWYNDLLVDLGSKDTANIYKGKKVDLYGVYYG      120
p_3      GSAIIENKESNDQFLKNTILFNDFFTGHQWYNDLLVDLGSKDTANIYKGKKVDLYGVYYG      120
         ************************************************************

p_4      YQCTGGTPFKTACMYGGVTLHDNNQLEEEKKVPINLWIDGKQNTVPLGTVKTNKKEVTVQ      180
p_2      YQCTGGTPFKTACMYGGVTLHDNNQLEEEKKVPINLWIDGKQNTVPLGTVKTNKKEVTVQ      180
p_3      YQCTGGTPFKTACMYGGVTLHDNNQLEEEKKVPINLWIDGKQNTVPLGTVKTNKKEVTVQ      180
         ************************************************************

p_4      ELELQSRHYLHETYNLYNTDAFNGKIQRGLIEFHPSSGDSVGYDLFGAQGQYPDTQLRIY      240
p_2      ELDLQSRHYLHETYNLYNTDAFNGKIQRGLIEFHPSSGDSVGYDLFGAQGQYPDTQLRIY      240
p_3      ELDLQSRHYLHETYNLYNTDAFNGKIQRGLIEFHPSSGDSVGYDLFGAQGQYPDTQLRIY      240
         **:*********************************************************

p_4      RDNKTIKSKNMHIDIYLYTT     260
p_2      RDNKTIKSKNMHIDIYLYTT     260
p_3      RDNKTIKSKNMHIDIYLYTT     260
         ********************
```

SEQ

```
q_3      MNKIFRILTVSLFFFTFLIKNNLAYADVGVINLRNFYANYEPEKLQGVSSGNFSTSHQLE      60
q_5      MNKIFRVLTVSLFFFTFLIKNNLAYADVGVINLRNFYTNYQPETLQGVSSGNFSTSHQLE      60
q_2      MNKIFRVLTVSLFFFTFLIKNNLAYADVGVINLRNFYANYQPEKLQGVSSGNFSTSHQLE      60
q_1      MNKIFRILTVSLFFFTFLIKNNLAYADVGVINLRNFYANYEPEKLQGVSSGNFSTSHQLE      60
q_4      MNKIFRILTVSLFFFTFLIKNNLAYADVGVINLRNFYANYEPEKLQGVSSGNFSTSHQLE      60
         ******:******************************:**:**.****************

q_3      YIDGKYTLYSQFHNEYEAKRLKDHKVDIFGISYSGLCNTKYMYGGITLANQNLDKPRNIP      120
q_5      YIDGKYTLYSQFHNEYEAKRLKDHKVDIFGISYSGLCNTKYMYGGITLANQNLDKPRNIP      120
q_2      YIDGKYTLYSQFHNEYEAKRLKDHKVDIFGISYSGLCNTKYMYGGITLANQNLDKPRNIP      120
q_1      YIDGKYTLYSQFHNEYEAKRLKDHKVDIFGISYSGLCNTKYMYGGITLANQNLDKPRNIP      120
q_4      YIDGKYTLYSQFHNEYEAKRLKDHKVDIFGISYSGLCNTKYMYGGITLANQNLDKPRNIP      120
         ************************************************************
```

```
q_3        INLWVNGKQNTISTDKVSTQKKEVTAQEIDIKLRKYLQNEYNIYGFNKTKKGQEYGYQSK        180
q_5        INLWVNGKQNTISTDEVSTQKKEVTAQEIDIKLRKYLQNEYNIYGFNKTKKGQEYGYQSK        180
q_2        INLWVNGKQNTISTDKVSTQKKEVTAQEIDIKLRKYLQNEYNIYGFNKTKKGQEYGYKSK        180
q_1        INLWVNGKQNTISTDKVSTQKKEVTAQEIDIKLRKYLQNEYNIYGFNKTKKGQEYGYQSK        180
q_4        INLWVNGKQNTISTDKVSTQKKEVTAQEIDIKLRKYLQNEYNIYGFNKTKKGQEYGYQSK        180
           **************:******************************************:**

q_3        FNSGFNKGKITFHLNNEPSFTYDLFYTGTGQAESFLKIYEDNKTVESEKFHLDVDISYKE        240
q_5        FNSGFNKGKITFHLNNETSFTYDLFYTGTGQAESFLKIYNDNKTIDAENFHLDVEISYEK        240
q_2        FNSGFNKGKITFHLNNEPSFTYDLFYTGTGQAESFLKIYNDNKTIDAENFHLDVEISYEK        240
q_1        FNSGFNKGKITFHLNNEPSFTYDLFYTGTGQAESFLKIYDDNKTIDTENFHLDVEISYEK        240
q_4        FNSGFNKGKITFHLNNEPSFTYDLFYTGTGQAESFLKIYNDNKTIDAENFHLDVEISYEK        240
           ***************** *********************:****:::*:*****:***::

q_3        TI      242
q_5        TE      242
q_2        TE      242
q_1        TE      242
q_4        TE      242
           *
```

SER

```
r_2        MLNKILLLLLFSVTFMLLFFSLHSVSAKPDPRPGELNRVSDYKKNKGTMGNVESLYKDKAV        60
r_5        MLNKILLLLLLSVTFMLLFFSLHSVSAKPDPRPGELNRVSDYKKNKGTMGNVESLYKDKAV        60
r_1        MLNKILLLLLLSVTFMLLFFSLHSVSAKPDPRPGELNRVSDYKKNKGTMGNVESLYKDKAV        60
r_4        MLNKILLLLLLSVTFMLLFFSLHSVSAKPDPRPGELNRVSDYKKNKGTMGNVESLYKDKAV        60
           *********:**************************************************

r_2        IAENVKNTRQFLGHDLIFPIPYSEYKEVKSEFINKKTADKFKDKRLDVFGIPYFYTCLVP        120
r_5        IAENVKNTRQFLGHDLIFPIPYSEYKEVKSEFINKKTADKFKDKRLDVFGIPYFYTCLVP        120
r_1        IAENVKNTRQFLGHDLIFPIPYSEYKEVKSEFINKKTADKFKDKRLDVFGIPYFYTCLVP        120
r_4        IAENVKNTRQFLGHDLIFPILYSEYKEVKSEFINKKTADKFKDKRLDVFGIPYFYTCLVP        120
           ******************* ****************************************

r_2        KNESREEFIFDGVCIYGGVTMHSTADSISKNIIVPVTVDNKQQFSFTISTNKKTVTVQEL        180
r_5        KNESREEFIFDGVCIYGGVTMHSTADSISKNIIVPVTVDNKQQFSFTISTNKKTVTVQEL        180
r_1        KNESREEFIFDGVCIYGGVTMHSTADSISKNIIVPVTVDNKQQFSFTISTNKKTVTVQEL        180
r_4        KNESREEFIFDGVCIYGGVTMHSTADSISKNIIVPVTVDNKQQFSFTISTNKKTVTVQEL        180
           ************************************************************

r_2        DYKVRNWLTNNKKLYEFDGSAYETGYIKFIEENKDSFWYDLFPKKDLVPFIPYKFVNIYG        240
r_5        DYKVRNWLTNNKKLYEFDGSSYETGYIKFIEENKDSFWYDLFPKKDLVPFIPYKFVNIYG        240
r_1        DYKVRNWLTNNKKLYEFDGSAYETGYIKFIEENKDSFWYDLFPKKDLVPFIPYKFVNIYG        240
```

```
r_4       DYKVRNWLTNNKKLYEFDGSAYETGYIKFIEENKDSFWYDLFPKKDLVPFIPYKFVNIYG        240
          *******************:****************************************

r_2       DNKTIDASSVKIEVHLTTM      259
r_5       DNKTIDASSVKIEVHLTTT      259
r_1       DNKTIDASSVKIEVHLTTT      259
r_4       DNKTIDASSVKIEVHLTTT      259
          ******************
```

SES

```
s_3       MNYSKITVSLIIIILLCTFSFEFSFNRFVQADESRPKIESLKKKSELDSTALYNIKTSYSQ        60
s_1       MNYSKITVTLIIIILLCTFSFEFSFNRFVQADESRPKIESLKKKSELDSTALYNIKTSYSQ        60
          ********:***************************************************

s_3       DNIILDIKNKTNSTQLLSNDLIFDDITLKEWNKNSLKTEFNSSEIANHFKGKKVDIFGIY        120
s_1       DNIILDIKNKTNSTQLLSNDLIFDDITLKEWNKNSLKTEFNSSEIANHFKGKKVDIFGIY        120
          ************************************************************

s_3       YGANCIGEVSKRTGCIYGGITLHEEEKIDQKNSIGVNVFKDGSQQKGFMITTDKKEPTIQ        180
s_1       YGANCIGEVSKRTGCIYGGITLHEEEKIDQKNSIGVNVFKDGSQQKGFMITTDKKEPTIQ        180
          ************************************************************

s_3       ELDLKTRKVIQNQYKIYNSETGNIQKGYMEFHSNSSSFYYDLFNFKGKYSVDFLKFYNDN        240
s_1       ELDLKTRKVIQNQYKIYNSETGNIQKGYMEFHSNSSSFYYDLFNFKGKYSVDFLKFYNDN        240
          ************************************************************

s_3       KTINSSNLHIDVYLYSQ       257
s_1       KTINSSNLHIDVYLYSQ       257
          *****************
```

SET

>t_1

```
MKKCILFIFSIILLVSISTLFIPNAKSDSREGLKDFYSKKIDVYTNKKINEKDKYSVDIEVDNYVYRINTLDDKILNQFKV
GDYVDAWGHIINNKPIGKVIKFYDGDISKHSPLDKPTNISYRINLFKEQKQTEVTPEKELEIGNRYLTMKQIDYRIKSYLV
KKQQLYTEFYNGKIEISMLDGKKHFIDLSTYYYDPSNFTLDYTKISHFDIYMEK
```

SEU

```
u_11      MKLFAFLFICVKSCSLLFMLNDNPKPEQLNKASEFTGLMDNMRYLYDDKHVSETNIKAQE        60
u_18      MKLFAFLFICVKSCSLLFMLNDNPKPEQLNKASEFTGLMDNMRYLYDDKHVSETNIKAQE        60
u_6       MKLFAFIFICVKSCSLLFMLNGNPKPEQLNKASEFTGLMDNMRYLYDDKHVSETNIKSQE        60
u_4       MKLFAFIFICVKSCSLLFMLNGNPKPEQLNKASEFTGLMDNMRYLYDDKHVSETNIKSQE        60
u_5       MKLFAFIFICVKSCSLLFMLNGNPKPEQLNKASEFTGLMDNMRYLYDDKHVSETNIKSQE        60
u_1       MKLFAFIFICVKSCSLLFMLNGNPKPEQLNKASEFTGLMDNMRYLYDDKHVSEINIKAQE        60
u_7       MKLFAFIFICVKSCSLLFMLNGNPRPEQLNKASEFSGLMDNMRYLYDDKHVSETNIKVQE        60
u_17      MKLFAFIFICVKSCSLLFMLNGNPRPEQLNKASEFSGLMDNMRYLYDDKHVSETNIKAQE        60
u_9       MKLFAFIFICVKSCSLLFMLNGNPRPEQLNKASEFTGLMDNMRYLYDDKHVSETNIKAQE        60
u_2       MKLFAFIFICVKSCSLLFMLNGNPRPEQLNKASEFTGLMDNMRYLYDDKHVSETNIKAQE        60
u_10      MKLFAFIFICVKSCSLLFMLNGNPRPEQLNKASEFTGLMDNMRYLYDDKHVSETNIKAQE        60
          ******:**************.**:***********:**************** *** **

u_11      KFLQHDLLFKINR-----SKILKTEFNNKSLSDKYKNKNVDLFGTNYYNQCYFSSDNMEL        115
u_18      KFLQHDLLFKING-----SKILKTEFNNKSLSDKYKNKNVDLFGTNYYNQCYFSADNMEL        115
u_6       KFLQHDLLFEING-----SKILKTEFNNKSLSDKYKNKNVDLFGTNYYNQCYFSSDNMEL        115
u_4       KFLQHDLLFKING-----SKILKTEFNNKSLSDKYKNKNVDLFGTNYYNQCYFSLDNMEL        115
u_5       KFLQHDLLFKING-----SKILKTEFNNKSLSDKYKNKNVDLFGTNYYNQCYFSSDNMEL        115
u_1       KFLQHDLLFKINGSKIDGSKILKTEFNNNSLSDKYKNKNIDLFGTNYYYQCYFSADNMEL        120
u_7       KFLQHDLLFKINGSKIDGSKILKTEFNNKSLSDKYKNKNVDLFGTNYYNQCYFSADNMEL        120
u_17      KFLQHDLLFKINGSKIDGSKILKTEFNNKSLSDKYKNKNVDLFGTNYYNQCYFSADNMEL        120
u_9       KFLQHDLLFKINGSKIDGSKILKTEFNNKSLSDKYKNKNVDLFGTNYYNQCYFSADNMEL        120
u_2       KFLQHDLLFKINGSKIDGSKILKTEFNNKSLSDKYKNKNVDLFGTNYYNQCNFSADNMEL        120
```

```
u_10      KFLQHDLLFKINGSKIDGSKILKTEFNNKSLSDKYKNKNVDLFGTNYYNQCYFSADNMEL      120
          *********:**      **********:**********:******** ** ** *****

u_11      NDGILIEKTCMYGGVTEHDGNQIDKNNSTDNSHNILIKVYENERNSLSFDIPTNKKNITA      175
u_18      NDGILIGKTCMYGGVTEHDGNQIDKNNSTDNSHNILIKVYENERNSLSFDIPTNKKNITA      175
u_6       NDGRLIEKTCMYGGVTEHDGNQIDKNNSTDNSHNILIKVYENERNSLSFDIPTNKKNITA      175
u_4       NDGRLIEKTCMYGGVTEHDGNQIDKNNSTDNSHNILIKVYENERNSLSFDIPTNKKNITA      175
u_5       NDGRLIEKTCMYGGVTEHDGNQIDKNNSTDNSHNILIKVYENERNSLSFDIPTNKKNITA      175
u_1       NDGRLIEKTCMYGGVTEHDGNQIDKNNSTDNSHNILIKVFENERNSLSFDIPTNKKNITA      180
u_7       NDGRLIEKTCMYGGVTEHDGNQIDKNNLTDNSHNILIKVYENERNTLSFDISTNMKNITA      180
u_17      NDGRLIEKTCMYGGVTEHDGNQIDKNNLTDNSHNILIKVYENERNTLSFDISTNMKNITA      180
u_9       NDGRLIEKTCMYGGVTEHDGNQIDKNNLTDNSHNILIKVYENERNTLSFDISTNKKNITA      180
u_2       NDGRLIEKTCMYGGVTEHDGNQIDKNNLTDNSHNILIKVYENERNTLSFDISTNKKNITA      180
u_10      NDGRLIEKTCMYGGVTEHDGNQIDKNNLTDNSHNILIKVYENERNTLSFDISTNKKNITA      180
          *** ** ******************** ***********:*****:***** ** *****

u_11      QEIDYKVRNYLLKHKDLYEFNSSPYETGYIKFIEGNGNTFWYDMMPESGEKFYPTKYLLI      235
u_18      QEIDYKVRNYLLKHKDLYEFNSSPYETGYIKFIEGNGNTFWYDMMPESGEKFYPTKYLLI      235
u_6       QEIDYKVRNYLLKHKNLYEFNSSPYETGYIKFIEGSGHSFWYDLMPESGKKFYPTKYLLI      235
u_4       QEIDYKVRNYLLKHKNLYEFNSSPYETGYIKFIEGSGHSFWYDLMPESGKKFYPTKYLLI      235
u_5       QEIDYKVRNYLLKHKNLYEFNSSPYETGYIKFIEGSGHSFWYDLMPESGKKFYPTKYLLI      235
u_1       QEIDYKVRNYLLKHKNLYEFNSSPYETGYIKFIEGNGHSFWYDMMPESGEKFYPTKYLLI      240
u_7       QEIDYKVRNYLLKHKNLYEFNSSPYESGYIKFIEGNGHSFWYDMMPESGEKFYPTKYLLI      240
u_17      QEIDYKVRNYLLKHKNLYEFNSSPYESGYIKFIEGNGHSFWYDMMPESGEKFYPTKYLLI      240
u_9       QEIDYKVRNYLLKHKNLYEFNSSPYETGYIKFIEGNSHSFWYDMMPESGEKFYPTKYLLI      240
u_2       QEIDYKVRNYLLKHKNLYKFNSSPYETGYIKFIEGNGHSFWYDMMPESGEKFYPTKYLLI      240
u_10      QEIDYKVRNYLLKHKNLYKFNSSPYETGYIKFIEGNGHSFWYDMMPESGEKFYPTKYLLI      240
          ***************:**:*******:********..::****:*****:**********

u_11      YNDNKTVDSQSVNVEVHLTKK   256
u_18      YNDNKTVDSQSVNVEVHLTKK   256
u_6       YNDNKTVESKSINVEVHLTKK   256
u_4       YNDNKTVESKSINVEVHLTKK   256
u_5       YNDNKTVESKSINVEVHLTKK   256
u_1       YNDNKTVESKSINVEVHLTKK   261
u_7       YNDNKTVESKSINVEVHLTKK   261
u_17      YNDNKTVESKSINVEVHLTKK   261
u_9       YNDNKTVESKSINVEVHLTKK   261
u_2       YNDNKTVESKSINVEVHLTKK   261
u_10      YNDNKTVESKSINVEVHLTKK   261
          *******:*:*:*********
```

SEX

```
x_27      MFKKHHSKNSIVLKSILSLGIIYSGIFGINSKADASIQDSSSVHDKQLLKVKEVPNNSEK      60
x_28      MFKKHHSKNSIVLKSILSLGIIYSGIFGINSKADASIQDSSSVHDKQLLKVEEVPNNSEK      60
x_47      MFKKHHSKNSIVLKSILSLGIIYSGIFGINSKADASIQDSSSVHDKQFQKVEEVPNNSEK      60
x_10      MFKKHHSKNSILLKSILSLGIIYSGSFGIYSKANASTQNSSSVHDKQLQKVEEVPNNSEK      60
x_34      MFKKHHSKNSILLKSILSLGIIYSGSFGIYSKANASTQNSSSVHDKQLQKVEEVPNNSEK      60
x_3       MFKKNDSKNSILLKSILSLGIIYGGTFGIYPKADASTQNSPSVQDKQFQKVEEVPNNSEK      60
x_16      MFKKNDSKNSILLKSILSLGIIYGGTFGIYPKADASTQNSPSVQDKQFQKVEEVPNNSEK      60
x_14      MFKKYGSKNSIVLKSILSLGIIYGGTFGIYPKADASTQNSSSVQDKQFQKVEEVPNNSEK      60
x_12      MFKKYDSKNSIVLKSILSLGIIYGGTFGIYPKADASTQNSSSVQDKQFQKVEEVPNNSEK      60
x_22      MFKKYDSKNSIVLKSILSLGIIYGGTFGIYPKADASTQNSSSVQDKQFQKVEEVPNNSEK      60
x_41      MFKKNDSKNSIVLKSILSLGIIYGGTFGIYPKADASTQNSSSVQDKQLQKVEEVPNNSEK      60
x_6       MFKKHHSKNSIVLKSILSLGIIYGGTFGIYPKADASTQNSSSVQDKQLQKVEEVPNNSEK      60
x_36      MFKKNDSKNSIVLKSILSLGIIYGGTFGIYPKADASTQNSSSVQDKQLQKVEEVPNNSEK      60
x_4       MFKKYDSKNSIVLKSILSLGIIYGGTFGIYPKADASTQNSSSVQDKQFQKVEEVPNNSEK      60
x_7       MFKKYDSKNSIVLKSILSLGIIYGGTFGIYPKADASTQNSSSVQDKQFQKVEEVPNNSEK      60
x_17      MFKKYDSKNSIVLKSILSLGIIYGGTFGIYPKADASTQNSSSVQDKQFQKVEEVPNNSEK      60
x_15      MFKKHHSKNSIVLKSILSLGIIYGGTFGIYPKADASTQNSSSVQDKQFQKVEEVPNNSEK      60
x_45      MFKKYDSKNSIVLKSILSLGTIYGGTFGIYPKADASTQNSSSVQDKQLQKVEEVPNNSEK      60
x_5       MFKKYDSKNSIVLKSILSLGIIYGGTFGIYPKADASTQNSSSVQDKQLQKVEEVPNNSEK      60
```

```
x_9         MFKKYDSKNSIVLKSILSLGIIYGGTFGIYPKADASTQNSSSVQDKQLQKVEEVPNNSEK       60
x_25        MFKKYDSKNSIVLKSILSLGIIYSGSFGIYPKADASTQNSSSVQDKQLQKVEEVPNNSEK       60
x_1         MFKKYDSKNSILLKSILSLGIIYGGTFGIYPKADASTQNSSSVQDKQLQKVEEVPNNSEK       60
x_24        MFKKYDSKNSIVLKSILSLGIIYGGTFGIYPKADASTQNSSSVQDKQLQKVEEVPNNSEK       60
            ****  *****:******** **.* ***  **:** *:* **:***: **:********

x_27        ALVKKLYDRYSQNTINGKSNKSRNWVYSERPLNENQVRIHLEGTYTVAGRVYTPKRNITL       120
x_28        ALVKKLYDRYSKDTINGKSNKSRNWVYSERPLNENQVRIHLEGTYTVAGRVYTPKRNITL       120
x_47        ALVKKLYDRYSQNTINGKSNKSRNWVYSERPLNENQVRIHLEGTYTVAGRVYTPKRNITL       120
x_10        ALVKKLYDRYSKNTINGKSNKSRNWVYSERPLNGNQVRINLEGTYRVADRVYTPKRNITL       120
x_34        ALVKKLYDRYSKNTINGKSNKSRNWVYSERPLNGNQVRIHLEGTYTVAGRVYTPKRNITL       120
x_3         ALVKKLYDRYSQNTINGKSNKSRNWVYSERPLNENQVRINLEGTYRVADRVYTPKRNITL       120
x_16        ALVKKLYDRYSQNTINGKSNKSRNWVYSERPLNENQVRINLEGTYRVADRVYTPKRNITL       120
x_14        ALVKKLYDRYSQNTINGKSNKARNWVYSERPLNENQVRIHLEGTYRVADRVYTPKRNITL       120
x_12        ALVKKLYDRYSQNTINGKSNKARNWVYSERPLNENQVRIHLEGTYRVADRVYTPKRNITL       120
x_22        ALVKKLYDRYSQNTINGKSNKARNWVYSERPLNENQVRIHLEGTYRVADRVYTPKRNITL       120
x_41        ALVKKLYDRYSQNTINGKSNKSRNWVYSERPLNENQVRIHLEGTYRVADRVYTPKRNITL       120
x_6         ALVKKLYDRYSKDTINGKSNKSRNWVYSERPLNENQVRIHLEGTYRVADRVYTPKRNITL       120
x_36        ALVKKLYDRYSKDTINGKSNKSRNWVYSERPLNENQVRIHLEGTYRVADRVYTPKRNITL       120
x_4         ALVKKLYDRYSQNTINGKSNKSRNWVYSERPLNENQVRIHLEGTYTVAGRVYTPKRNITL       120
x_7         ALVKKLYDRYSQNTINGKSNKSRNWVYSERPLNENQVRIHLEGTYTVADRVYTPKRNITL       120
x_17        ALVKKLYDRYSQNTINGKSNKSRNWVYSERPLNENQVRIHLEGTYTVADRVYTPKRNITL       120
x_15        ALVKKLYDRYSKNTINGKSNKSRNWVYSERPLNGNQVRIHLEGTYTVAGRVYTPKRNITL       120
x_45        ALVKKLYDRYSKDTINGKSNKSRNWVYSERPLNENQVRIHLEGTYTVAGRVYTPKRNITL       120
x_5         ALVKKLYDRYSKDTINGKSNKSRNWVYSERPLNENQVRIHLEGTYTVAGRVYTPKRNITL       120
x_9         ALVKKLYDRYSKDTINGKSNKSRNWVYSERPLNENQVRIHLEGTYTVADRVYTPKRNITL       120
x_25        ALVKKLYDRYSKDTINGKSNKSRNWVYSERPLNENQVRIHLEGTYTVAGRVYTPKRNITL       120
x_1         ALVKKLYDRYSKDTINGKSNKSRNWVYSERPLNENQVRIHLEGTYTVAGRVYTPKRNITL       120
x_24        ALVKKLYDRYSKDTINGKSNKSRNWVYSERPLNENQVRIHLEGTYTVAGRVYTPKRNITL       120
            ***********::********:*********** *****:***** **.***********

x_27        NKEVVTLKELDHIIRFAHISYGLYMGEHLPKGNIVINTKNGGKYTLESHKELQKDRENVK       180
x_28        NKEVVTLKELDHIVRFAHISYGLYMGEHLPKGNIVINTKDGGKYTLESHKELQKDRENVE       180
x_47        NKEVVTLKELDHIVRFAHISYGLYMGEHLPKGNIVINTKDGGKYTLESHKELQKDRENVE       180
x_10        NKEVVTLKELDHIIRFAHISYGLYMGEHLPKGNIVINTKDGGKYTLESHKELQKDRENVK       180
x_34        NKEVVTLKELDHIIRFAHISYGLYMGEHLPKGNIVINTKNGGKYTLESHKELQKDRENVK       180
x_3         NKEVVTLKELDHIIRFAHISYGLYMGEHLPKGNIVINTKDGGKYTLESHKELQKDRENVK       180
x_16        NKEVVTLKELDHIIRFAHISYGLYMGEHLPKGNIVINTKDGGKYTLESHKELQKDRENVK       180
x_14        NKEVVTLKELDHIIRFAHISYGLYMGEHLPKGNIVINTKNGGKYTLESHKELQKNRGNVE       180
x_12        NKEVVTLKELDHIIRFAHISYGLYMGEHLPKGNIVINTKNGGKYTLESHKELQKNRENVE       180
x_22        NKEVVTLKELDHIIRFAHISYGLYMGEHLPKGNIVINTKNGGKYTLESHKELQKNRENVE       180
x_41        NKEIVTLKELDHIIRFAHISYGLYMGEHLPKGNIVINTKNGGKYTLESHKELQKDRENVK       180
x_6         NKEVVTLKELDHIIRFAHISYGLYMGEHLPKGNIVINTKNGGKYTLESHKELQKDRENVK       180
x_36        NKEVVTLKELDHIIRFAHISYGLYMGEHLPKGNIVINTKNGGKYTLESHKELQKDRENVK       180
x_4         NKEVVTLKELDHIIRFAHISYGLYMGEHLPKGNIVINTKNGGKYTLESHKELQKDRENVK       180
x_7         NKEVVTLKELDHIIRFAHISYGLYMGEHLPKGNIVINTKNGGKYTLESHKELQKDRENVK       180
x_17        NKEVITLKELDHIIRFAHISYGLYMGEHLPKGNIVINTKNGGKYTLESHKELQKDRENVK       180
x_15        NKEVVTLKELDHIIRFAHISYGLYMGEHLPKGNIVINTKNGGKYTLESHKELQKNRENVE       180
x_45        NKEVVTLKELDHIIRFAHISYGLYMGEHLPKGNIVINTKDGGKYTLESHKELQKDRENVK       180
```

```
x_5          NKEVVTLKELDHIIRFAHISYGLYMGEHLPKGNIVINTKDGGKYTLESHKELQKDRENVK       180
x_9          NKEVVTLKELDHIIRFAHISYGLYMGEHLPKGNIVINTKDGGKYTLESHKELQKDRENVK       180
x_25         NKEVVTLKELDHIIRFAHISYGLYMGEHLPKGNIVINTKNGGKYTLESHKELQKNRENVE       180
x_1          NKEVVTLKELDHIIRFAHISYGLYMGEHLPKGNIVINTKNGGKYTLESHKELQKNRENVE       180
x_24         NKEVVTLKELDHIIRFAHISYGLYMGEHLPKGNIVINTKNGGKYTLESHKELQKNRENVE       180
             ***::********:*************************:**************:* **:

x_27         INTDDIKNVTFELVKSVNDIEQA 203
x_28         INTADIKNVTFELVKSVNDIEQA 203
x_47         INTADIKNVTFELVKSVNDIEQA 203
x_10         INTADIKNVTFKLVKSVNDIEQV 203
x_34         INTDDIKNVTFELVKSVNDIEQV 203
x_3          INTADIKNVTFKLVKSVNDIEQV 203
x_16         INTTDIKNVTFKLVKSVNDIEQV 203
x_14         INTDDIKNVTFELVKSVNDIEQV 203
x_12         INTDDIKNVTFELVKSVNDIEQV 203
x_22         INTDDIKNVTFELVKSVNDIGQV 203
x_41         INTDDIKNVTFELVKSVNDIEQV 203
x_6          INTDDIKNVTFELVKSVNDIEQV 203
x_36         INTDDIKNVTFELVKSVNDIEQV 203
x_4          IKTDDIKNVTFELVKRVNDIEQV 203
x_7          NNTDDIKNVTFELVKSVNDIEQV 203
x_17         INTDDIKNVTFELVKSVNDIEQV 203
x_15         INTADVENVTFDLVKSVNDIEQV 203
x_45         INTADIKNVTFKLVKSVNDIEQV 203
x_5          INTADIKNVTFKLVKSVNDIEQV 203
x_9          INTADIKNVTFKLVKSVNDIEQV 20
x_25         INTDDIKNVTFELVKSVNDIEQV 203
x_1          INTDDIKNVTFELVKSVNDIEQV 203
x_24         INTDDIKNVTFELVKSVNDIEQV 203
              :* *::****.*** **** *.
```

SEY

```
y_5          MKAKLWFLLTTLAFLIAVMGSIGIAEVGAKTTGLITENSNDSLKEHYAQKFEVYTNKEVT       60
y_11         MKAKLWFLLTTLAFLIAVMGSIGIAEVEAKTTGLITENSNDSLKEHYAQKFEVYTNKEVT       60
y_2          MKAKLCFLLTTLAFLIAVTGSIGIAEVKAKTTGLITENSNDSLKEHYAQKFEVYTNKEVT       60
y_1          MKAKLWFLLTTLAFLIAVTGSIGIAEVKAKTTGLITENSNDSLKEHYAQKFEVYTNKEIT       60
y_7          MKAKLWFLLTTLAFLIAVTGSIGIAEVKAKTTGLITENSNDSLKEHYAQKFEVYTNKEVT       60
y_6          MKAKLWFLLTTLAFLIAVTGSIGIAEVKAKTTGLITENSNDSLKEHYAQKFEVYTNKEVT       60
y_3          MKAKLWFLLTTLAFLIAVTGSIGIAEVKAKTTGLITENSNDSLKEHYAQKFEVYTNKEVT       60
y_4          MKAKLWFLLTTLAFLIAVTESIGIAEVKAKTTGLITENSNDSLKEHYAQKFEVYTNKEVT       60
             ***** ************  ******* ******************************:*

y_5          GVGENYIDAKVDTYNVRTVLYNTDYLKQFKNKDKVNIWGTLYENQQSKVYRGTVVKYDPI       120
y_11         GVGENYIDAKVDTYNVRTVLYNTDYLKQFKNKDKVNIWGTLYENQQSKVYRGTVVKYDPI       120
y_2          GVGENYIDAKVDTYNVRTVLYNTDYLKQFKNQDKVNIWGTLYENQQSKVYRGTVVKYDPI       120
y_1          GVGENYIDAKVDTYNVRTVLYNTDYLKQFKNQDKVNIWGTLYENKQSKVYRGTVVKYDPI       120
y_7          GVGENYIDAKVDTYNVRTVLYNTDYLKQFKNQDKVNIWGTLYENQQSKVYRGTVVKYDPI       120
y_6          GVGENYIDVKVDTYNVRTVLYNTDYLKQFKNQDKVNIWGTLYENQQSKVYRGTVVKYDPI       120
y_3          GVGENYIDTKVDTYNVRTVLYNTDYLKQFKNQDKVNIWGTLYENQQSKVYRGTVVKYDPI       120
```

```
y_4         GVGENYIDTKVDTYNVRTVLYNTDYLKQFKNQDKVNIWGTLYENQQSKVYRGTVVKYDPI      120
            ********.**********************:************:***************

y_5         SKVTNLSYRMNLFVNGHQTKVNPDSLLEVKNKQISLKETDFRIRKYLLEKEHLYSNYNSG      180
y_11        SKVTNLSYRMNLFVNGHQTKVNPDSLLEVKNKQISLKETDFRIRKYLLEKEHLYSNYNSG      180
y_2         SKVTNLSYRMNLFVNGHQTKVNPDSLLEVKNKQISLKETDFRIRKYLLEKEHLYSNYNSG      180
y_1         SKVTNLSYRMNLFVNGHQTKVNPDSLLEVKNKQISLKETDFRIRKYLLEKEHLYSNYNSG      180
y_7         SKVTNLSYRMNLFVNGHQTKVNPDSLLEVKNKQISLKETDFRIRKYLLEKEHLYSNYNSG      180
y_6         SKVTNLSYRMNLFVNGHQTKVNPDSLLEVKNKQISLKETDFRIRKYLLEKEHLYSNYNSG      180
y_3         SKVTNLSYRMNLFVNGHQTKVNPDSLLEVKNKQISLKETDFRIRKYLLEKEHLYSNYNSG      180
y_4         SKVTNLSYRMNLFVNGHQTKVNPDSLLEVKNKQISLKETDFRIRKYLLEKEHLYSNYNSG      180
            ************************************************************

y_5         ELIIEMKNGTRHKIDLGDILSDSQEKTFDFDNISHIDIYMK        221
y_11        ELIIEMKNGTRHKIDLGDILSDSQEKTFDFDNISHIDIYMK        221
y_2         ELIVEMKNGARHKIDLGDILSDSQEKTFDFDNISHIDIYMK        221
y_1         ELIIEMKNGARHKIDLGDILSDSQEKTFDFDNISHIDIYMK        221
y_7         ELIIEMKNGARHKIDLGDILSDSQEKTFDFDNISHIDIYMK        221
y_6         ELIIEMKNGARHKIDLGDILSDSQEKTFDFDNISHIDIYMK        221
y_3         ELIIEMKNGARHKIDLGDILSDSQEKTFDFDNISHIDIYMK        221
y_4         ELIIEMKNGARHKIDLGDILSDSQEKTFDFDNISHIDIYMK        221
            ***:*****:*******************************
```

SEZ

```
z_5        MRKVFILITLLFGYSSYSLLQAKAETQNDPNISELNKSSQYTGSWHNIWYLYNSDPVNAK      60
z_3        MRKVFILITLLFGYSSYSLLEARAETQNDPNISELNKSSQYTGSWHNIWYLYNSDPVNAK      60
z_1        MRKIFILITLLFGYSSYSLLEARAETQNDPNISELNKSSQYTGSWHNIWYLYNSDPVNAK      60
z_2        MRKVFILITLLFGYSSYSLLEARAETQNDPNISELNKSSQYTGSWHNIWYLYNSDPVNAK      60
           ***:****************:*:*************************************

z_5        KIKLSDKFLSHDFIVPINSPGHYDYVKTELKDSTMASSFDGKEVDIFGVNYFDQCYFSNE      120
z_3        KIKLSDKFLSHDFIVPINNPGHYDYVKTELKDSTMASSFDGKEVDIFGVNYFDQCYFSNE      120
z_1        KIKLSDKFLSHDFIVPINNPGHYDYVKTELKDSTMASSFDGKEVDIFGVNYFDQCYFSNE      120
z_2        KIKLSDKFLSHDFIVPINNPGHYDYVKTELKDSTMASSFDGKEVDIFGVNYFDQCYFSNE      120
           ******************.*****************************************

z_5        NIQCDSNQGAGSKKTCMYGGITLNENNTNNRIQPIVVKVYENDSVTLSFDINIDKETVTI      180
z_3        NIQCDSNQGAGSKKTCMYGGITLNENNTNNRIQPIVVKVYENDSVTLSFDINIDKETVTI      180
z_1        NIQCDSNQGGGSKKTCMYGGITLNENNTNNRIQPIVVKVYENDSVTLSFDINIDKETVTI      180
z_2        NIQCDSNQGGGSKKTCMYGGITLNENNTNNRIQPIVVKVYENDSVTLSFDINIDKETVTI      180
           *********.**************************************************

z_5        QELDYKVRNKLISKINLYHLGGTSYETGYIKFIENGNRYYWYDMMPDPGFTQSKYLMIYR      240
z_3        QELDYKVRNKLISKINLYHLGGTSYETGYIKFIENGNRYYWYDMMPDPGFTQSKYLMIYR      240
z_1        QELDYKVRNKLISKINLYHLGGTSYETGYIKFIENGNRYYWYDMMPDPGFTQSKYLMIYR      240
z_2        QELDYKVRNKLISKINLYHLGGTSYETGYIKFIENGNRYYWYDMMPDPGFTQSKYLMIYR      240
           ************************************************************
```

```
z_5       GNETVESAKTEIEVHLTKK      259
z_3       GNETVESAKTEIEVHLTKK      259
z_1       GNETVESAKTEIEVHLTKK      259
z_2       GNETVESAKTEIEVHLTKK      259
          *******************
```

TSST1

```
TSST1_6       MNKKLLMNFFIVSPLLLATIATDFTPVPLSSNQIIKTAKASTNDNIKDLLDWYSSGSDTF   60
TSST1_1       MNKKLLMNFFIVSPLLLATIATDFTPVPLSSNQIIKTAKASTNDNIKDLLDWYSSGSDTF   60
TSST1_3       MNKKLLMNFFIVSPLLLATIATDFTPVPLLSNQIIKTAKASTNDNIKDLLDWYSSGSDTF   60
TSST1_5       MNKKLLMKFFIVSPLLLATIATGFTPVPLSSNQIIKTAKASTNDNIKDLLDWYSSGSDAF   60
TSST1_2       MNKKLLMKFFIVSPLLLATIATGFTPVPLSSNQIIKTAKASTNDNIKDLLDWYSSGSDAF   60
TSST1_4       MNKKLLMKFFIVSPLLLATIATGFTPVPLSSNQIIKTAKASTNDNIKDLLDWYSSGSDAF   60
              *******:**************.****** ****************************:*

TSST1_6       TNSEVLDNSLGSMRIKNTDGSISLIIFPSPYYSPAFTKGGKVDLNTKRTKKSQHTSEGTY   120
TSST1_1       TNSEVLDNSLGSMRIKNTDGSISLIIFPSPYYSPAFTKGEKVDLNTKRTKKSQHTSEGTY   120
TSST1_3       TNSEVLDNSLGSMRIKNTDGSISLIIFPSPYYSPAFTKGEKVDLNTKRTKKSQHTSEGTY   120
TSST1_5       TNSEVLDNSLGSMRIKNTDGSISLIIFPSPYYSPTFSKGEKVDLNTKRTKKSQHTSEGTW   120
TSST1_2       TNSEVLDNSLGSMRIKNTDGSISLIIFPSPYYSPTFSKGEKVDLNTKRIKKSQHTSEGTW   120
TSST1_4       TNSEVLDNSLGSMRIKNTDGSISLIIFPSPYYSPTFSKGEKVDLNTKRIKKSQHTSEGTW   120
              **********************************:*:** ******** **********:

TSST1_6       IHFQISGVTNTEKLPTPIELPLKVKVHGKDSPLKYWPKFDKKQLAISTLDFEIRHQLTQI   180
TSST1_1       IHFQISGVTNTEKLPTPIELPLKVKVHGKDSPLKYWPKFDKKQLAISTLDFEIRHQLTQI   180
TSST1_3       IHFQISGVTNTEKLPTPIELPLKVKVHGKDSPLKYWPKFDKKQLAISTLDFEIRHQLTQI   180
TSST1_5       IHFQISGVTNTEKLPTPIELPLKVKVHGKDSPLKYWPKFDKKQLAISTLDFEIRHQLTQI   180
TSST1_2       IHFQISGVTNTEKLPTPIELPLKVKVHGKDSPLKYWPKFDKKQLAISTLDFKIRHQLTQT   180
TSST1_4       IHFQISGVTNTEKLPTPIELPLKVKVHGKNSPLKYWPKFDKKQLAISTLDFKIRHQLTQT   180
              *****************************:*********************:*******

TSST1_6       HGLYRSSDKTGGYWKITMNDGSTYQSDLSKKFEYNTEKPPINIDEIKTIEAEIN 234
TSST1_1       HGLYRSSDKTGGYWKITMNDGSTYQSDLSKKFEYNTEKPPINIDEIKTIEAEIN 234
TSST1_3       HGLYRSSDKTGGYWKITMNDGSTYQSDLSKKFEYNTEKPPINIDEIKTIEAEIN 234
TSST1_5       HGLYRSSDKTGGYWKITMNDGSTYQSDLSKKFEYNTEKPPINIDEIKTIEAEIN 234
TSST1_2       HGLYRSSDKTGGYWKITMNDGSTYQSDLSKKFEYNTEKPPINIDEIKTIEAEIN 234
TSST1_4       HGLYRSSDKTGGYWKITMNDGSTYQSDLSKKFEYNTEKPPINIDEIKTIEAEIN 234
              ******************************************************
```
